# Surveillance, insecticide resistance and control of an invasive *Aedes aegypti *(Diptera: Culicidae) population in California

**DOI:** 10.12688/f1000research.8107.3

**Published:** 2016-08-05

**Authors:** Anthony J. Cornel, Jodi Holeman, Catelyn C. Nieman, Yoosook Lee, Charles Smith, Mark Amorino, Katherine K. Brisco, Roberto Barrera, Gregory C. Lanzaro, F. Stephen Mulligan III

**Affiliations:** 1 Mosquito Control Research Laboratory, Kearney Agricultural Center, Department of Entomology and Nematology, UC Davis, Parlier, CA, USA; 2 Consolidated Mosquito Abatement District, Selma, CA, USA; 3 Vector Genetics Laboratory, School of Veterinary Medicine, UC Davis, Davis, CA, USA; 4 Entomology and Ecology Actvity7, Dengue Branch, Centers for Disease Control and Prevention, San Juan, Puerto Rico

**Keywords:** Aedes aegypti, surveillance, mosquito control, insecticide resistance, California, kdr

## Abstract

The invasion and subsequent establishment in California of *Aedes aegypti *in 2013 has created new challenges for local mosquito abatement and vector control districts. Studies were undertaken to identify effective and economical strategies to monitor the abundance and spread of this mosquito species as well as for its control. Overall, BG Sentinel (BGS) traps were found to be the most sensitive trap type to measure abundance and spread into new locations. Autocidal-Gravid-Ovitraps (AGO-B), when placed at a site for a week, performed equally to BGS in detecting the presence of female *Ae. aegypti.* Considering operational cost and our findings, we recommend use of BGS traps for surveillance in response to service requests especially in locations outside the known infestation area. We recommend AGO-Bs be placed at fixed sites, cleared and processed once a week to monitor mosquito abundance within a known infestation area. Long-term high density placements of AGO-Bs were found to show promise as an environmentally friendly trap-kill control strategy. California *Ae. aegypti *were found to be homozygous for the V1016I mutation in the voltage gated sodium channel gene, which is implicated to be involved in insecticide resistance. This strain originating from Clovis, California was resistant to some pyrethroids but not to deltamethrin in bottle bio-assays. Sentinel cage ultra-low-volume (ULV) trials using a new formulation of deltamethrin (DeltaGard®) demonstrated that it provided some control (average of 56% death in sentinel cages in a 91.4 m spray swath) after a single truck mounted aerial ULV application in residential areas.

## Introduction

*Aedes aegypti* Linnaeus is presumed to have become established in the southeastern United States of America between the fifteenth and eighteenth centuries ( Tabachnick, 1991). Its spread to the US between 1795 and 1905 precipitated major epidemics of yellow fever throughout the east coast and southern states ( Crosby, 2006). Today this mosquito serves as the vector of three additional human viruses, dengue, chikungunya and Zika, which pose a major threat to global public health.

The state of California, however, had remained free from this vector until the summer of 2013 ( Gloria-Soria *et al.*, 2014), when *Ae. aegypti* were simultaneously collected in CO _2_-Baited Encephalitis Virus Surveillance traps (EVS) in the cities of Clovis (Fresno County) and Madera (Madera County) and later in oviposition cups in Menlo Park (San Mateo County). Within three months, *Ae. aegypti,* immature and adults were detected within a 1.6 km radius around the initial collection site in Clovis. An interactive guide tracking the 2013 and 2014 progression of the *Ae. aegypti* invasion in Clovis can be viewed as a webpage story developed by J. Holeman ( http://bit.ly/1qB3CVD). Collections of *Ae. aegypti* in 2014 and 2015, and further expansion of its distribution proved that this mosquito species is capable of surviving through the winter and has established as a viable breeding population in California. By spring of 2015, this mosquito had been collected in seven additional California counties (Kern, Imperial, Los Angeles, Orange, Riverside, San Diego and Tulare). The ongoing widespread invasion and establishment of *Ae. aegypti* proves that this is a state-wide and not simply a regional issue in California.

Multiple control measures were immediately implemented by the Consolidated Mosquito Abatement District (CMAD) in response to the initial discovery of *Ae. aegypti* in the city of Clovis. Control efforts included: (i) thorough property inspection for potential larval development sites, (ii) sanitation, (iii) insecticide applications to larval sources, (iv) residual barrier spraying with pyrethroid insecticides and (v) public education. Public education included distribution of information packets (consult www.mosquitobuzz.net for content) to all households within 512 meters of a positive collection site. Television and internet broadcasts and press stories were released throughout the period from initial detection to present day to generate public awareness.

To validate and improve *Ae. aegypti* surveillance and control, CMAD selected four traps from the wide variety available. These included: (i) the CO _2_- baited EVS trap ( Rohe & Fall, 1979), (ii) oviposition cups ( Barbosa *et al.*, 2010; Furlow & Young, 1970), (iii) CO _2_- baited BG Sentinel (BGS) trap ( www.bg-sentinel.com- without octenol attractant) and (iv) Autocidal Gravid Oviposition trap (AGO-B) ( MacKay *et al.*, 2013). The decision to use CO _2_ with the BGS was based on a study by de Ázara *et al.* (2013) that showed that more females were collected in BGS baited with CO _2_ than without.

Pyrethroid insecticides are the preferred method to control adult mosquitoes in California. However, there are numerous reports of pyrethroid resistant *Ae. aegypti* populations worldwide ( Aponte *et al.*, 2013; Hemingway & Ranson, 2000; Martins *et al.*, 2009; Rawlins, 1998). Therefore the CMAD conducted insecticide susceptibility bio-assays on the Clovis population to determine their susceptibility to pyrethroids. In this study, bottle bio-assays were conducted exposing mosquitoes to pyrethrum, pyrethrum + piperonyl butoxide (PBO), permethrin, permethrin + PBO, sumithrin, deltamethrin and malathion. A portion of the voltage gated-sodium channel ( *vgsc*) gene was sequenced in several mosquitoes to determine if well-known insecticide resistant mutations were present in the California *Ae. aegypti*. These genotypes confer resistance to both DDT and pyrethroid insecticides ( Martins *et al.*, 2009; Saavedra-Rodriguez *et al.*, 2007). Sentinel cage mortality counts of *Ae. aegypti* were conducted in order to compare various ultra-low-volume (ULV) insecticide formulations. These involved the aerial delivery of insecticides from truck mounted sprays and were conducted in both open field and residential settings.

Integrated pest management (IPM) strategies that incorporate non-chemical based control methods are strongly recommended for mosquito control in California. Sustained high density placement of AGO-Bs, have shown promise as an effective *Ae. aegypti* trap-kill control measure in Puerto Rico ( Barrera *et al.*, 2014; MacKay *et al.*, 2013). As part of fulfilling the IPM mission this non-chemical based strategy (using low density placement of AGO-Bs) was evaluated in Clovis.

## Methods

### Trap method evaluation for surveillance purposes

**Figure 1.**
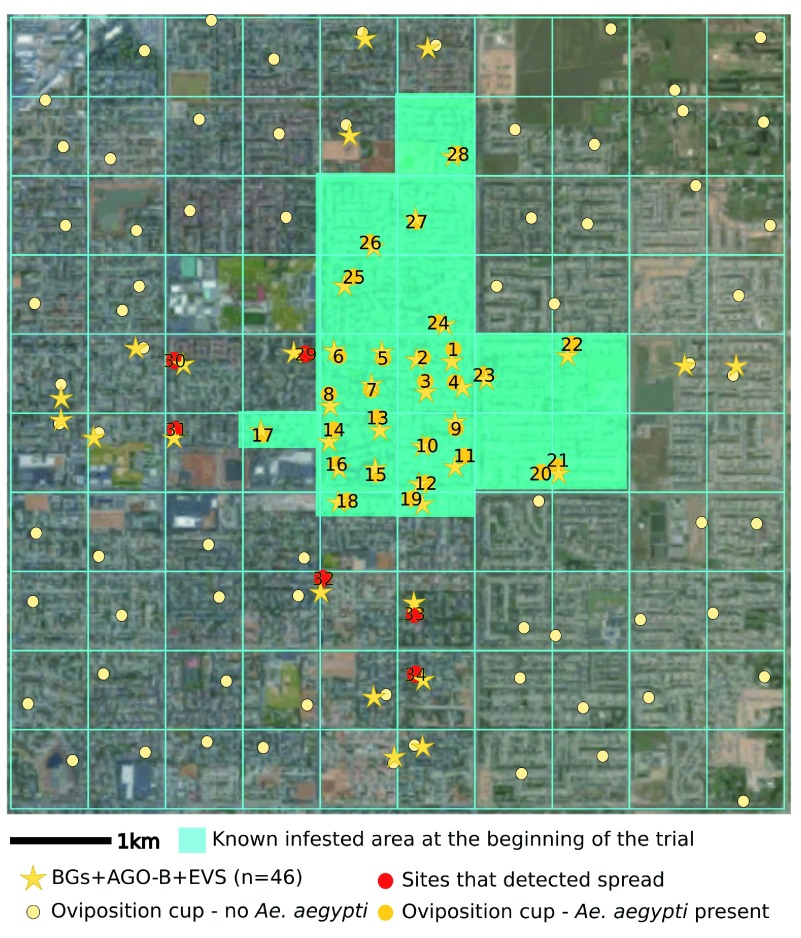
Layout of traps in a 10 × 10 cell grid covering 16.5 km ^2^ to evaluate trap types. Locations of oviposition cups are represented by dark circles and AGO-Bs as stars. The blue shaded area corresponds to the area where *Ae. aegypti* were present at the start of the evaluation (infestation area). Traps are marked in red circles where *Ae. aegypti* were collected after commencement of the study, showing dispersal. Numbers correspond to trap site locations. Detailed trap count data for each of the 34 sites that collected mosquitoes at least once during the trial are provided in Supplementary figures S1–S7.

A 10 × 10 cell grid covering 16.5 km ^2^ (each cell represented a 0.16 km ^2^) (center coordinates: 36.4845° N, 119.4006° W) was selected for this study during summer 2013. The grid incorporated *Ae. aegypti* infested and non-infested areas ( Figure 1). All four trap types were placed within the grid, which included 18 sites outside and 28 sites within the infestation area. The infestation area was defined as the area where *Ae. aegypti* had previously been recorded (within blue shaded area in Figure 1). The infestation area increased due to dispersal by the end of the study so that there were 34 positive trap sites in week 10.

One AGO-B and one oviposition cup were placed approximately 50 m apart in each front yard site. These two traps were left continuously operational. Adult mosquitoes were counted and removed once a week from the AGO-B trap. Oviposition cups were checked weekly for the presence of eggs and a new sheet of oviposition paper (germination paper, Seedburo Equipment Company, Chicago, IL) was added. Each week the AGO-B and oviposition cup location were switched at each site. One night per week (1:00 pm to 8:00 am) an EVS and a BGS trap were placed 50 m apart in a property adjacent to each yard that had an AGO-B and oviposition cup. The EVS and BGS traps were rotated between each other every week. Mosquito numbers in the AGO-B traps were divided by seven to facilitate comparison of average trap night count with the EVS and BGS traps. Trap evaluations continued for nine weeks and both males and females were counted in the adult traps.

Sites selected within the infestation area were used to determine which of the three adult collecting trap types consistently collected the greatest number of adults (marked in stars in Figure 1). The goal was to identify the trap type most sensitive for ongoing *Ae. aegypti* surveillance. Adult mosquitoes were counted and removed from the AGO-B once every 7 days and from BGS and EVS 24 hours after each deployment. The sites outside the infestation area were used to determine which of the four trap types was most effective at first detecting *Ae. aegypti* dispersing out of the infestation area and therefore could be used to track dispersal of this mosquito.

Comparisons in numbers of *Ae. aegypti* adults collected by the different adult trap types were calculated for significant differences using the Wilcoxon-Rank-sum test ( Bauer, 1972) implemented in the R statistical package version 3.0.0.

### Mosquito collections and colony maintenance for insecticide resistance and ULV trial evaluations

Larvae of *Ae. aegypti* reared from eggs collected in oviposition cups in Clovis, California (CLOVIS strain) were used for bottle bio-assays and ULV trials. They were reared on a diet of ground rodent chow at 27°C under 14:10 hour (light:dark) photoperiod and adults were held at 70% relative humidity. Adult mosquitoes had unlimited supply to a 10% sucrose solution and were human blood-fed by AJC. The Rockefeller (ROCK) strain ( Martins *et al.*, 2009) was used as the susceptible *Ae. aegypti* strain and were reared under the same conditions.

### Adult bottle bio-assays

Time to knockdown adulticide bottle bio-assays were conducted by treating the insides of 250 ml Wheaton bottles (Fisher #06-404B) with technical grade insecticides purchased from Chem Service (West Chester, PA). The insecticides were diluted in acetone and bottles were coated with the insecticide following the procedure described in Brogdon & McAllister (1998). For each insecticide, six replicates of 25 three to four day old adult mosquitoes were used to determine percentage mortality (malathion) and percent knock-down (pyrethroids) every 15 minutes for up to 2 hours and every 5 minutes between the 30 ^th^ and 45 ^th^ minutes. Control bottles were coated with acetone only. Mosquitoes that could not maintain an upright position when the bottle was rotated slowly were considered knocked down or dead. Mosquitoes were exposed to a predetermined dosage of insecticide that resulted in 100% mortality or knock-down within 30 minutes of the standard susceptible ROCK strain ( Kuno, 2010). All bio-assays on the Clovis population were run simultaneously with the control susceptible ROCK strain. Concentrations of insecticide each test bottle was coated with were: Malathion = 50μg/ml; Deltamethrin = 10μg/ml; Sumithrin = 20μg/ml; Pyrethrum = 15.6μg/ml; Permethrin = 15μg/ml. The pyrethrum consisted of 14.2% pyrethrin I isomer and 10.7% pyrethrin II isomer (Lot # 2693200) and permethrin isomer ratio was 75.1% TRANS and 24.6% CIS (Lot # 3565000). The 400μg PBO per bottle dose used with pyrethrum and permethrin was the maximum amount that did not cause mortality when used alone. For bio-assays that included PBO, the mosquitoes were first exposed to PBO for one hour and then transferred to bottles coated with the insecticide.

Knockdown time (KDT) or lethal time (LT) was calculated using a binary logistic regression implemented in the MASS library ( Ripley *et al.*, 2015) in R software package ( R Core Team). Significance testing comparing 50% knock-down time (KDT _50_) and 90% knock-down time (KDT _90_) between the ROCK and CLOVIS strains exposed to the different chemicals and with and without PBO within strains were performed by Wilcoxon Rank-sum test ( Bauer, 1972) using the R software package ( R Core Team).

### *vgsc* sequencing

The IIS5-S6 region of the voltage gated sodium channel ( *vgsc*) gene of 13 adult *Ae. aegypti* from Madera and 13 adults from Clovis, collected in BGS traps in the last week of August 2013, were sequenced using conventional Sanger Sequencing method. Samples were lysed using a Qiagen Tissulyser and genomic DNA extracted using a BioSprint 96 DNA Blood Kit (Qiagen, Chatsworth, CA) using the Qiagen BioSprint protocols described in Nieman *et al.* (2015). The PCR reaction was carried out following the protocol described in Martins *et al.* (2009). Amplicons were sequenced at the UC ^-^DNA Sequencing Facility (College of Biological Sciences, UC Davis) using an ABI 3730 Genetic Analyzer (Applied Biosystems, Carlsbad, California). Gene fragments were also sequenced in both directions (forward/reverse) and SNPs were identified only if the SNP was found in both directions. *Geneious* ( Kearse *et al.*, 2012) software version 6.1.4 was used for sequence alignment and SNP identification.

### Ultra-low volume (ULV) application trials

**Figure 2.**
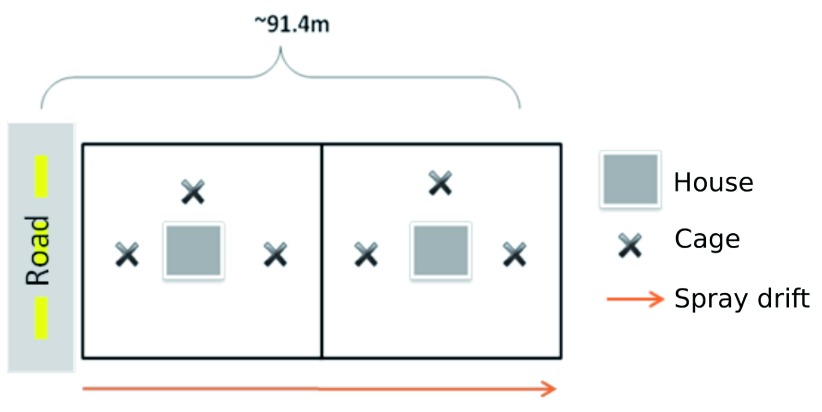
Placement of sentinel cages at Clovis residences to evaluate the control efficacy of aerial truck mounted ULV deltamethrin (DeltaGard®) application. Distance between the sentinel cages from the street to the furthest sentinel in the front yard of the next parallel street was 91.44 m.

Adult mosquitoes were exposed under operational field conditions to the following commercial ULV adulticide formulations; 6% pyrethrins, 60% piperonyl butoxide (PBO) (Evergreen EC® 60-6, MGK, Minneapolis, MN) (flow rate: 153.8 mL/min); etofenprox (Zenivex® E20, Wellmark International, Schaumburg, IL) (flow rate: 106.5 mL/min; and deltamethrin (DeltaGard®, Bayer, Research Triangle Park, NC) (flow rate: 356.4 mL/min). Evergreen EC® 60-6 and Zenivex® E20 are registered in California. DeltaGard® is not presently registered in California and a Research Authorization (approved RA-1505051) was obtained from the CA Department of Pesticide Regulation for evaluating this product for this study. Application rates and relevant meteorological conditions during applications of the three ULV trials are provided in Table 1. All three ULV applications were evaluated in a fallow open field (36.4941° N, 119.4441° W) and the trial using deltamethrin was also performed in a residential area within the city of Clovis (36.4801° N, 119.4003° W) where houses, various vegetation, and fences acted as barrier objects to the spray. Mosquito control efficacy results were based on 12 hour post exposure mortalities recorded in sentinel cages placed in rows perpendicular to the wind direction and downwind from the line of application. Approximately 20 CLOVIS and ROCK mosquitoes were placed in screened sentinel cages ( Townzen & Natvig, 1973) 3 to 6 hours prior to the ULV trial. Mosquitoes in the sentinel cages were provided access to a cotton swab soaked with a 10% sucrose solution and held in a cool environment in insulated boxes for transport to the field. Within 30 minutes prior to the commencement of the trial, sentinel cages were attached to stakes 1 m above ground. The stakes were placed in the ground 15.25, 30.48, 60.96 and 91.44 m downwind from an application in the open setting. Stakes holding sentinel cages in the residential setting were positioned in the configuration depicted in Figure 2. This configuration was designed to assess penetration of the ULV (91.4 m swath) in the urban residential. All applications were made with a truck mounted, cold aerosol ULV sprayer (Cougar model with SmartFlow, Clarke, Roselle, ILL) traveling at 16.1 km/h. Controls were placed in an area away from the spray sites. Sentinel cages were left on the stakes for an hour post application, after which knock-down and mortality was recorded in each cage. The mosquitoes were left in the cages, and each cage was covered on one side with a lightly dampened towel, the cotton swabs were re-soaked with 10% sucrose and each cage was individually placed into a plastic bag and held for a further 12 hours in insulated boxes. After 12 hours, mortality was recorded in each cage. Sentinel cages from the control sites were treated in exactly the same manner.

**Table 1.** Percent mortality recorded 12 hours after exposure to ULV insecticide in open and residential settings. Zenivex® was applied at 4g/ha, MGK Pyrocide® at 60g/ha and DeltaGard® at 1.5g/ha. A temperature inversion of 1°C and wind speeds of 2.41 to 4.83 km/hr were recorded during the open field applications. A temperature inversion of 0.3°C and wind speeds 8.85 to 12.55 km/hr were recorded during the trial in the Clovis residential area.

				Distance from application (m)						
Treatment		Strain	Trial(s)	15.24	30.48	60.96	91.44	Mean	Strain difference P-value	Distance linear model Slope (P-value)
Etofenprox ^a^	Open field	Clovis	1	76.5	85.7	57.1	73.3	74.6	0.00021	-0.0005 (P=0.78)
			2	90.0	52.9	81.0	80.0			
		Rock	1–2	100	100	100	100	100		-3.1x10 ^-18^ (P=0.27)
Pyrethrum + PBO ^b^	Open field	Clovis	1	100	35.7	68.4	64.7	58.4	0.00073	-0.0032 (P=0.45)
			2	93.3	11.1	69.2	25.0			
		Rock	1/2	100	100	100	100	100		-3.1x10 ^-18^ (P=0.27)
Deltamethrin ^c^	Open field	Clovis	1–2	100	100	100	100	100	NA	-3.1x10 ^-18^ (P=0.27)
		Rock	1–2	100	100	100	100	100		-3.1x10 ^-18^ (P=0.27)
		CQ1	1–2	100	100	100	100	100		-3.1x10 ^-18^ (P=0.27)
				Placement from application						
				Front	Middle		Back				
Deltamethrin ^d^	Residential	Clovis	1	48.5	21.7		26.3	57.3	6.26x10 ^-5^	0.00063 (P=1.00)
			2	73.1	42.3		33.3			
			3	100	84.0		100			
			4	28.9	37.9		91.4			
		Rock	1–4	100	100		100	100		

^a^ Zenivex®,;

^b^ MGK Pyrocide®,

^c and d^ DeltaGard®

Two glass microscope slides (Bioquip, Rancho Dominguez, CA) mounted on spinners (Hock Company, Gainesville, FL) adjacent to the sentinel cages were used to record droplet size and density of passing airborne spray across 91.44 m in both the open and residential settings. Teflon coated slides were used for all ULV trials except for the trials using deltamethrin which were coated with magnesium oxide. DropVision® was used as the software system to read the slides and generate the droplet analysis reports (Leading Edge Associates, LLC out of Waynesville, NC). Slides were digitally read using a specialized Motic DMBA300 Teflon slide reading compound microscope (Leading Edge Associates Inc.) at 100X magnification.

Regression line slope calculations were performed to examine if there was any difference in mortalities of mosquitoes placed at various distances from the spray sources to test for distance effect. Wilcoxon rank-sum tests were performed to compare mortalities of mosquitoes in sentinel cages across the 91.44 m swath for each mosquito strain exposed to each ULV formulation. Both the regression line slopes ( Chambers, 1992) and Wilcoxon rank-sum tests ( Bauer, 1972) were calculated using the corresponding option in the R software package.

### Autocidal Gravid Ovitrap (AGO-B) control evaluation

**Figure 3.**
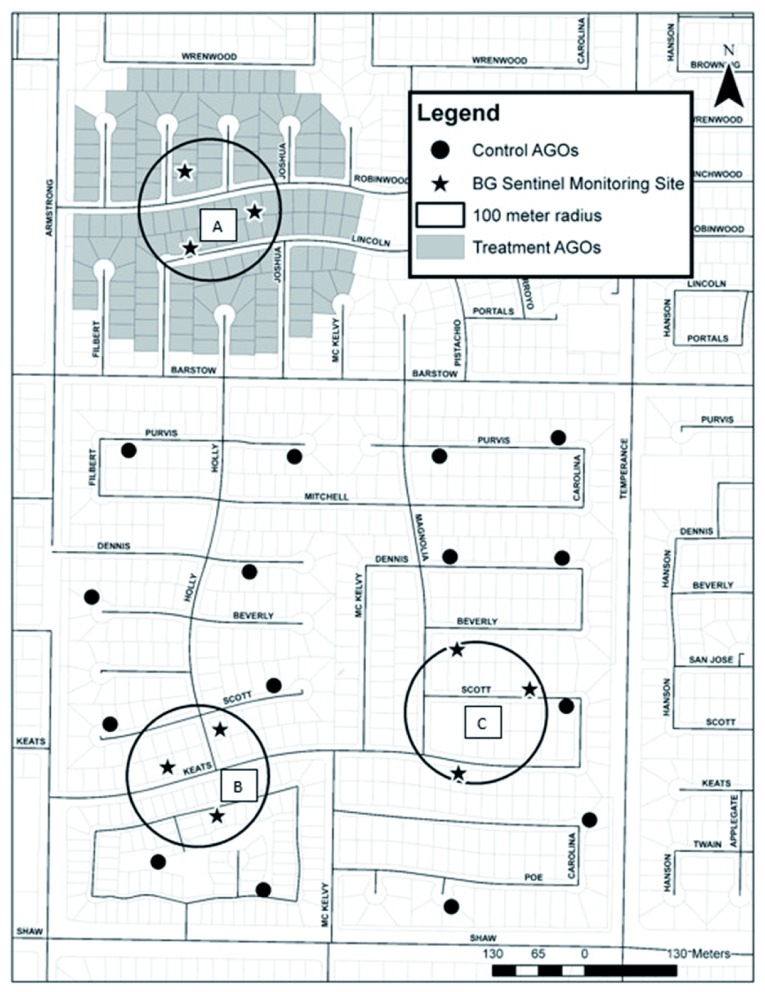
Map showing the layout and positions of AGO-B and BGS traps to evaluate the control efficacy of high density placement of AGO-B traps in residential Clovis. Properties shaded in grey were those that had an AGO-B trap placed in their front yard (144 traps in total). Stars represent locations of BGS traps used to measure abundance per week in both the control and intervention areas. Dark circles show positions of AGO-B traps outside the intervention area which were used for control site monitoring.

As a trap-kill system, the AGO-B was designed to capture female *Ae. aegypti* on a sticky surface as they entered the trap to oviposit ( Barrera *et al.*, 2014). To evaluate this trap-kill control concept, three general locations within the Clovis *Ae. aegypti* infestation area were selected ( Figure 3). Each of the three locations were more than 200 meters apart, a distance further than the typical distance *Ae. aegypti* fly (60–100 m, Harrington *et al.*, 2005; Valerio *et al.*, 2012). Within the intervention area (area A in Figure 3) (center coordinates: 36.4904° N, 119.4014° W), one AGO-B was placed in the front yard of each of 144 households. In this study, one AGO-B was placed at each parcel in contrast to three AGO-Bs per parcel in Barrera *et al.* (2014). Six BGS were deployed within the two control areas (areas B and C in Figure 3) (area B center coordinates: 36.4838° N, 119.4014° W) (area C center coordinates: 36.4841° N, 119.3959° W) and monitored for a 12 week period; two weeks before and four weeks after deployment of the AGO-Bs within area A (treatment site). Three BGS were also used to monitor mosquito numbers within the treatment site for 12 weeks. Trap counts from an additional fifteen AGO-Bs positioned outside the treatment area (dark circles in Figure 3) were also included in the study to represent control area female AGO-B counts. Female mosquitoes were counted in AGO-Bs once a week in the treatment and control areas, and male and female *Ae. aegypti* were counted in BGS twice a week for the duration of the trial.

Relative temporal abundance comparisons of *Ae. aegypti* in the AGO-Bs and BGS traps between the treatments (area A) and control sites (areas B and C), were calculated as normalized proportions per week per area, by dividing the number of mosquitoes trapped per week by the total number of mosquitoes collected from the corresponding trap over the six weeks. Normalizing done to BGS trap counts in the control areas B and C were combined. Regression line slopes and statistical significance were calculated using a linear model function, lm ( Wilkinson & Rogers, 1973), in the R statistics package.

## Results

Figures S1–S11 for ‘Surveillance, insecticide resistance and control of an invasive *Aedes aegypti* (Diptera: Culicidae) population in California’ Click here for additional data file. Copyright: © 2016 Cornel AJ et al. 2016 Data associated with the article are available under the terms of the Creative Commons Zero "No rights reserved" data waiver (CC0 1.0 Public domain dedication).

Raw data for ‘Surveillance, insecticide resistance and control of an invasive *Aedes aegypti* (Diptera: Culicidae) population in California’ Includes collection counts ('Counts.csv'), knockdown and morality responses ('Summary.csv' and 'Knockdown-Mortality.csv'), and relative abundances (‘Abundance.csv’) of *Ae. aegypti*. Click here for additional data file. Copyright: © 2016 Cornel AJ et al. 2016 Data associated with the article are available under the terms of the Creative Commons Zero "No rights reserved" data waiver (CC0 1.0 Public domain dedication).

### Trap efficacy

**Figure 4.**
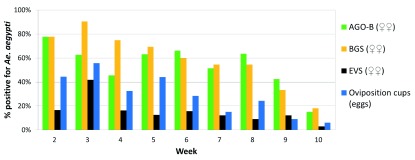
Trap efficacy measured by the percentage of sites positive for female *Ae. aegypti* (eggs for the case of the oviposition cups) per trap type in each of the ten weeks of the study. Data included the 34 traps numbered in Figure 1.

Numbers of males and females collected in the various trap types within the infestation area varied considerably from week to week ( Dataset 1). Mean numbers and SD of *Ae. aegypti* collected at each site in each trap is given above each bar in Figures S1 to S7 ( Dataset 1). Overall the BGS traps collected the largest number of adult *Ae. aegypti* and these were collected at significantly (P<0.005) more sites than either the AGO-B or EVS traps. However, the AGO-B and BGS traps performed equally, with no significant difference (P>0.05), in detection of female *Ae. aegypti* ( Figure 4, Wilcoxon rank sum test P-value=0.23). BGS traps collected more males than AGO-B traps in most weeks (Figures S2 and S4, Dataset 1).

### Detecting dispersal

Of the 18 sites outside the infestation area, only six recorded the presence and therefore spread of *Ae. aegypti* during the 10 week trial period. In three of these sites both AGO-B and BGS traps collected mosquitoes in the same week. In the other three sites only the BGS collected *Ae. aegypti.* None of the EVS and oviposition cup traps outside the infestation area collected *Ae. aegypti* adults or eggs. The sites that captured mosquitoes outside the original infestation area are represented by the red circles in Figure 1.

### Insecticide resistance genetics

The DNA fragment containing the IIS5-S6 region of *vgsc* had identical nucleotide sequences among all samples from Madera and Clovis (GenBank accession: KU728155-6). See Dataset 1 for sequence and alignment to the Liverpool strain reference sequence. All Madera (N=13) and Clovis (N=13) *Ae. aegypti* were homozygous for the known pyrethroid resistant V1016I mutation. The intron between exons 20 and 21 of California *Ae. aegypti* were 15 bp longer than the reference genome with 73% sequence similarity to the reference strain. In addition to the sequence difference in V1016I (exon 21), there were two other synonymous nucleotide differences in exons 20 between the California mosquitoes and the reference genomes (amino acid position 981 and 982).

### Insecticide resistance bio-assays

**Figure 5.**
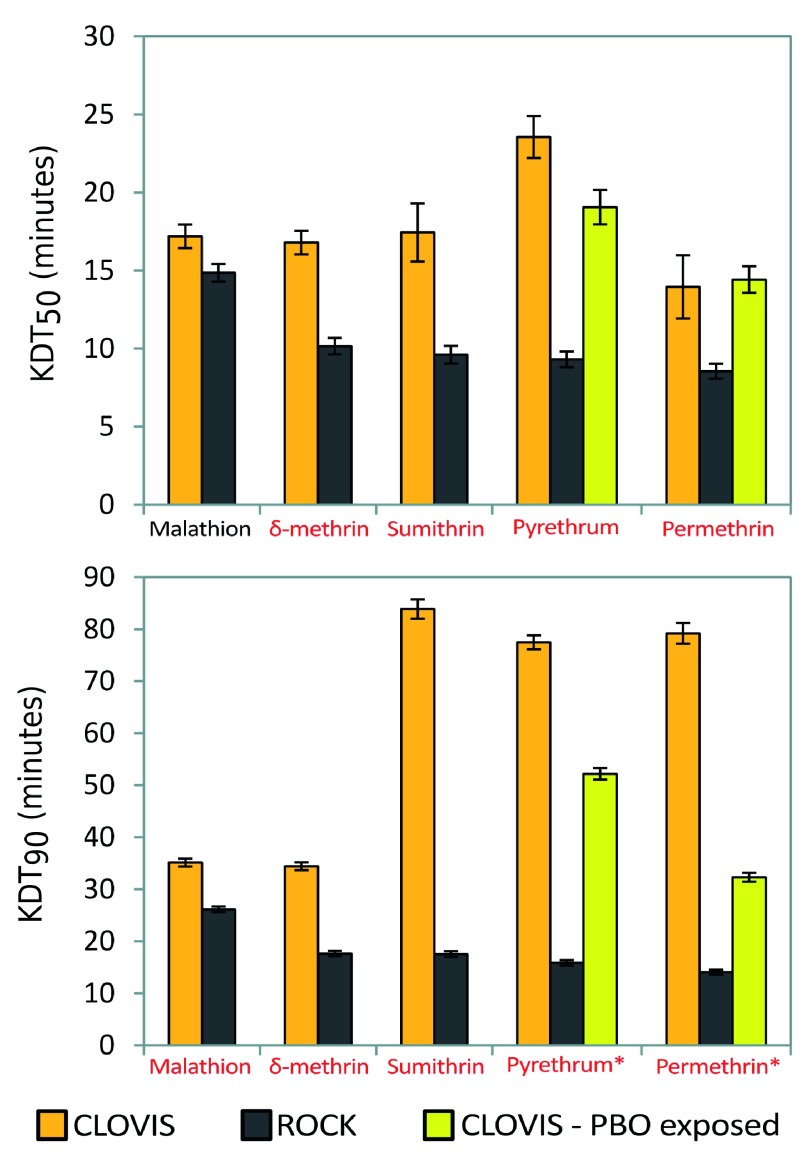
Box plot representation of median knock-down time (KDT _50_ and KDT _90_) and lethal time (LT _50_ and LT _90_) responses of *Ae. aegypti* in bottle bio-assays to various insecticides with and without PBO. ROCK refers to the susceptible Rockefeller colony strain and CLOVIS refers to mosquitoes found in Clovis. Concentrations of insecticide each test bottle was coated with were: Malathion = 50μg/ml; Deltamethrin = 10μg/ml; Sumithrin = 20μg/ml; Pyrethrum = 15.6μg/ml; Permethrin = 15μg/ml. Significant differences in knock-down time or mortality between the CLOVIS and ROCK strains is indicated by red text (Wilcoxon rank sum test α<0.05 or P<0.0032). * Significant differences in knock-down time distribution between treatment with and without PBO (Wilcoxon rank sum test α<0.05 or P < 0.0032).

In the standard bottle bio-assays no mortality was observed with either strain in the control bottles. Also no mortality was observed in mosquitoes due to PBO exposure for one hour before placement into bottles coated with the pyrethroids. The KDT _50_ and KDT _90_ times for the CLOVIS strain were highly variable between the six bottle replicates coated with sumithrin and pyrethrum and to a lesser extent with permethrin ( Figure 5) but were still significantly longer than the ROCK strain (P=0.0032). Exposing the CLOVIS mosquitoes to PBO for one hour significantly narrowed and slightly shortened their KDT _90_ to times closer to that experienced by the ROCK strain ( Figure 5). Both the CLOVIS and ROCK strains produced similar knock-down times and mortality against deltamethrin and malathion respectively ( Figure 5). The raw data is provided in Supplemental Dataset 2.

### ULV trials

Less than 10% mortality occurred in sentinel cages positioned in the up-wind control site locations for all trials. Mortality in sentinels exposed to insecticides was corrected for natural mortality by Abbott’s formula ( Abbott, 1925). No insecticide droplets were observed on the slides at the control sites in the open and residential ULV trials. The average droplet size and distribution recorded at all distances in ULV trials fell within the recommended range of 5–25μm for ground ULV applications ( Bonds, 2012).

There was no significant decline in mortality over distance from spray source (linear model P>0.05; Table 1) in sentinel caged mosquitoes in any of the open field ULV trials. Because there was no significant mortality effect in distance from spray source within the swath (91.44 m) we combined mortalities in all sentinels to produce a single average mortality across the swath. Applications of pyrethrum + PBO, etofenprox and deltamethrin in open settings resulted in 100% mortality at all distances from the spray source up to 91.44 m in the susceptible ROCK ( *Ae. aegypti)* mosquito strain ( Table 1). The mortality rate in the CLOVIS strain was significantly lower than the ROCK strain for both etofenprox (Wilcoxon rank sum test P=0.00021) and pyrethrum + PBO (P=0.00073) applications ( Table 1). In the open field ULV deltamethrin application 100% mortality was achieved with both the CLOVIS and ROCK strains, confirming the bottle bio-assay data that Clovis *Ae. aegypti* were susceptible to deltamethrin. In the ULV trial conducted in the residential area, there was also no significant decline in mortality over distance from spray source in sentinel caged mosquitoes ( Table 1). However, the CLOVIS strain had significantly lower mortality than the ROCK strain (Wilcoxon rank-sum test P=6.26×10 ^-5^). At the dosage of deltamethrin applied, which was less than half of the maximum allowable, according to the label, mortality of the Clovis *Ae. aegypti* was at an average rate of 55.64% along the full swath length.

### AGO-B trial

**Figure 6.**
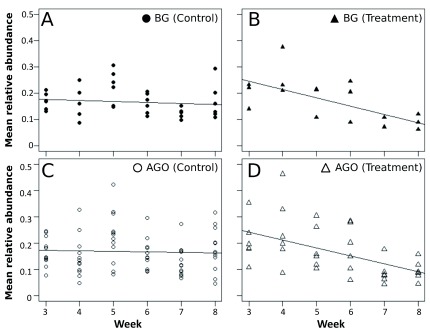
Relative abundance of *Ae. aegypti* in BGS and AGO-B traps in the control and treatment areas during the 6 week period of AGO-B trap deployment. Relative abundance of females and males were calculated in BGS traps and only females in AGO-B traps.

Counts of *Ae. aegypti* males and females collected in BGS and AGO-B traps in the treatment and control sites are provided in Figures S8–S10 ( Dataset 1). During the 12 week study period in 2014, the six BGS traps in the control areas collected 650 males (mean= 5.75 [SD=6.54] per trap night) and 1035 females (mean= 9.16 [SD=6.55] per trap night). The 15 control AGO-B traps collected 1,189 females (mean = 6.6 [SD=6.2] per week). During this six week period when the AGO-B traps were deployed, there was no significant decline in *Ae. aegypti* collected in the BGS traps (slope= -0.0041; P=0.46) or AGO-B traps (slope= -0.0019; P=0.72) within the control areas ( Figure 6A and 6C). However, during the same period, a decline in *Ae. aegypti* counts in BGS traps in the intervention area was significant (slope= -0.0315; P=0.0029; Figure 6B) and a decline in *Ae. aegypti* in the AGO-B traps in the intervention area was significant (slope= -0.0298; P=0.00087; Figure 6D). The raw data is provided in Supplemental Dataset 3.

## Discussion

### Trap type evaluation

During the dry summer breeding period (May–October) in Clovis, the major source of water to sustain breeding of *Ae. aegypti* is water accumulation in small containers and refuse from residential watering. In other dry urban locations where *Ae. aegypti* is found, such as in Arizona, watering by homeowners and monsoonal summer rainfall create sources of water for breeding. Based on the oviposition cup data within the infestation area during the 10 week trap evaluation trial the average numbers of eggs deposited in oviposition cups in Clovis per week was 291 (SD=432.6), which was less than the average of 447.6 eggs/day in Tucson, Arizona ( Hoeck *et al.*, 2003). The average number of female *Ae. aegypti* in BGS traps per night were 4 individuals in the primary Clovis infestation area and a similar abundance was observed between the summer months (June–September) of 2013 and 2014. Average BGS trap counts in Clovis were similar to the average of 4.67 per BGS trap night counts in Cairns, Australia ( Williams *et al.*, 2006) which were both lower than the average per night trap count of 58.8 females in BGS traps in Florida ( Wright *et al.*, 2015). Average numbers of female *Ae. aegypti* per week in AGO-B traps in Puerto Rico of 3.83 ( Barrera *et al.*, 2014) is higher than the average numbers collected in Clovis which was 2 mosquitoes per week in the 34 traps deployed during the 10 week trap evaluation trial in 2013 and about 2.75 mosquitoes per week in the 15 AGO-Bs deployed in the control areas during the 12 week AGO-B control evaluation trial in 2014.

Despite the uniform residential setting in Clovis, variable temporal abundance of adult *Ae. aegypti* was observed in this study regardless of trap type (Figure S1, Dataset 1). The highly variable spatial and temporal numbers of mosquitoes collected in BGS traps in Clovis is typical for *Ae. aegypti* trapping dynamics in general ( Degener *et al.*, 2014; Williams *et al.*, 2007). This clustering and variation in numbers needs to be taken into account by public health and mosquito control agencies when monitoring abundance over time, even in relatively small areas. In this study, the spatial design of trap placements was not appropriate to measure clustering and aggregate effects of *Ae. aegypti* in Clovis. However, clustering of *Ae. aegypti* typically does occur in residential areas ( Williams *et al.*, 2006) and this also needs to be considered in the design of monitoring strategies. Williams *et al.* (2007) recommended use of square-root transformations rather than log data transformations to deal with non-normally distributed BGS trap count data.

In this study, BGS traps out-performed the other three trap types in measuring both the spread and abundance of *Ae. aegypti* in Clovis. However, purchase and operational costs of BGS traps and homeowner cooperation in placement of traps must be considered in trap selection and use. Consequently, the CMAD now utilizes the general surveillance strategy described as follows: BGS traps are deployed in response to public service requests, particularly in locations outside known infestation areas to get a quick but sensitive measure of *Ae. aegypti* presence and to document dispersal. To correct for daily fluctuations in trap collections that could cause a missed detection of *Ae. aegypti*, AGO-B traps are also deployed at some properties outside the known infestation areas. AGO-B traps are deployed for a week or longer as opposed to BGS traps which are set out for only one day. Some AGO-B traps are also placed at fixed sites to monitor general abundance within the known infestation area. As an augmentative measure, oviposition cups, which are less expensive and less time consuming to utilize, are used at fixed points both within and outside the infestation area. Deployment of oviposition cups is often less conspicuous and may generate less homeowner concern and greater acceptance.

### Insecticide resistance and control considerations

The bottle bio-assay data provided clear evidence that the Clovis *Ae. aegypti* population is resistant to some pyrethroids such as permethrin, sumithrin and pyrethrum. All the Clovis and Madera *Ae. aegypti* sequenced (N=26), were fixed for the V1016I amino acid substitution which is one of the knock-down resistance mutations in *vgsc* responsible for reduced sensitivity to pyrethroids ( Saavedra-Rodriguez *et al.*, 2007). Multiple amino acid substitutions, associated with pyrethroid resistance, clustered within the II24-S5 linker, 11S5-S6 helices and the corresponding regions of domain III of the sodium channel gene ( Vontas *et al.*, 2012) have been found in various populations of *Ae. aegypti* worldwide, and they also include other mutations such as V1016G ( Brengues *et al.*, 2003; Chang *et al.*, 2009) and F1534C ( Harris *et al.*, 2010). Presence of V1016I mutation in Mexican *Ae. aegypti* populations ( Vera-Maloof *et al.*, 2015) add support toward the argument that Mexico may be a possible source of the founding populations of *Ae. aegypti* in California. Another possible origin of California *Ae. aegypti* may be the southeastern United States as suggested by Gloria-Soria *et al.* (2014) microsatellite analysis.

Addition of PBO in bottle bio-assays reversed resistance to pyrethrum significantly (P<0.003; Figure 5), suggesting that some P450s were additionally responsible for conferring metabolic resistance to pyrethrum in Clovis *Ae. aegypti.* The addition of PBO to permethrin did not significantly reduce knock-down time ( Figure 5). The wide range of knock-down times in response to permethrin and pyrethrum exposures indicate that the detoxifying role of P450s was variable between individuals and hence is likely a genetically polymorphic trait among Clovis *Ae. aegypti.*

Interestingly, despite being fixed for the V1016I mutation and having indications of the presence of the P450 metabolic pathway, the Clovis *Ae. aegypti* were not resistant to the pyrethroid deltamethrin. Presence of the V1016G and F1534C substitutions and other metabolic mechanisms associated with pyrethroid resistance in *Ae. aegypti* ( Vontas *et al.*, 2012) have yet to be found in the *Ae. aegypti* introduced into California.

Increased susceptibility to pyrethrum by addition of PBO warranted evaluating the control efficacy of a synergized pyrethrum + PBO formulation in a field ULV trial situation. In the open line application with no obstruction to the material drift, all the ROCK strain died but only 57.9% of CLOVIS were killed in sentinel cages within a 91.44 m swath (Table 2). The low mortality of Clovis *Ae. aegypti* in the ULV trial was unexpected because there was a strong synergizing effect observed in the bottle bio-assays ( Figure 5). Pyrthrum + PBO formulations are favored for ULV control in California because of labeling which allows application over agricultural crops. Unfortunately, results from this study indicate that pyrethrum + PBO ULV formulations may not control *Ae. aegypti* in Clovis.

Vertebrate toxicity effects of PBO are of concern to the public and two other ULV pyrethroid formulations with no PBO were evaluated in field ULV trials. Higher mortality was achieved with etofenprox in the Clovis *Ae. aegypti* in an open ULV trial (75%). Bottle bio-assays were not performed with etofenprox because specific crystallization properties of this chemical prevent it from coating surfaces evenly, and an even coating of the bottles is required for consistency of bottle bio-assay results. High mortality of Clovis *Ae. aegypti* (100%) was achieved with deltamethrin (DeltaGard®) in the open ULV trial, as was expected due to supportive low knock-down times observed in the bottle bio-assays ( Figure 5). Application of DeltaGard® in a residential Clovis setting resulted in lower but still promising 57.3% mortality of sentinel caged Clovis *Ae. aegypti* after exposure. Based on these results, we believe that use of this formulation may be effective to achieve significant immediate suppression of adult females in disease epidemic situations when applied in multiple consecutive nights as recommended by Macedo *et al.* (2010). Differences in mortality in Clovis *Ae. aegypti* between open and residential applications were likely due to reduced spread and penetration of the aerosolized product around residential structures and landscapes and less optimal local meteorological conditions. The most preferable time for ULV applications in the southern San Joaquin Valley of California is generally at sunset, when temperature inversions and wind conditions are most favorable for achieving the required 91.44 m swath insecticide drift. However, this is a peak time of day for human activity in residential areas. Timing of application may prove somewhat of a limiting factor for routine use of ground based adulticide application efforts against *Ae. aegypti.*

### Control by AGO-Bs

We observed a gradual decline in *Ae. aegypti* counts in areas where a single AGO-B trap was deployed at every household as an intervention. These results suggest that long-term, high density placement of AGO-B traps could be effective in Clovis. We speculate that deployment of multiple (3–4) AGO-B traps per parcel, similar to Barrera *et al.* (2014), might reduce *Ae. aegypti* populations below nuisance or disease transmission levels.

## Conclusion

We provided much needed information regarding the effective and economical strategies of surveillance and control for the Zika and other arbovirus vector, *Ae. aegypti.* Considering operational cost and our control research results, we recommend use of BGS traps for surveillance for *Ae. aegypti* in locations where presence of *Ae. aegypti* has not been recorded. AGO-Bs can be used as a surveillance tool within a known infestation area. Long-term high density placements of AGO-Bs were found to show promise as an environmentally friendly trap-kill control strategy. We recommend conducting insecticide resistance assays of *Aedes aegypti* populations wherever they exist because their susceptibility to insecticides differ geographically. Our surveillance and control methods can be applied to other closely related species such as *Aedes albopictus* which also transmits arboviruses and share similar biology. Given that *Ae. aegypti* transmits multiple serious viral diseases to humans, it is strongly recommended to include mosquito control research to monitor and develop effective control strategies.

## Data availability

The data referenced by this article are under copyright with the following copyright statement: Copyright: © 2016 Cornel AJ et al.

Data associated with the article are available under the terms of the Creative Commons Zero "No rights reserved" data waiver (CC0 1.0 Public domain dedication). 

*F1000Research*: Figures S1–S11 for 'Surveillance, insecticide resistance and control of an invasive *Aedes aegypti* (Diptera: Culicidae) population in California', 10.5256/f1000research.8107.d114301 ( Cornel *et al*., 2016a).

*F1000Research*: Dataset 2. Raw data for ‘Surveillance, insecticide resistance and control of an invasive *Aedes aegypti* (Diptera: Culicidae) population in California’, 10.5256/f1000research.8107.d115636 ( Cornel *et al.*, 2016b).
