# Supplementary material for: Surveillance, insecticide resistance and control of an invasive Aedes aegypti (Diptera: Culicidae) population in California
Source: F1000Res. 2016 Aug 5;5:194. Originally published 2016 Feb 19. [Version 3] doi: 10.12688/f1000research.8107.3 (PMC4857756; doi:10.12688/f1000research.8107.3)

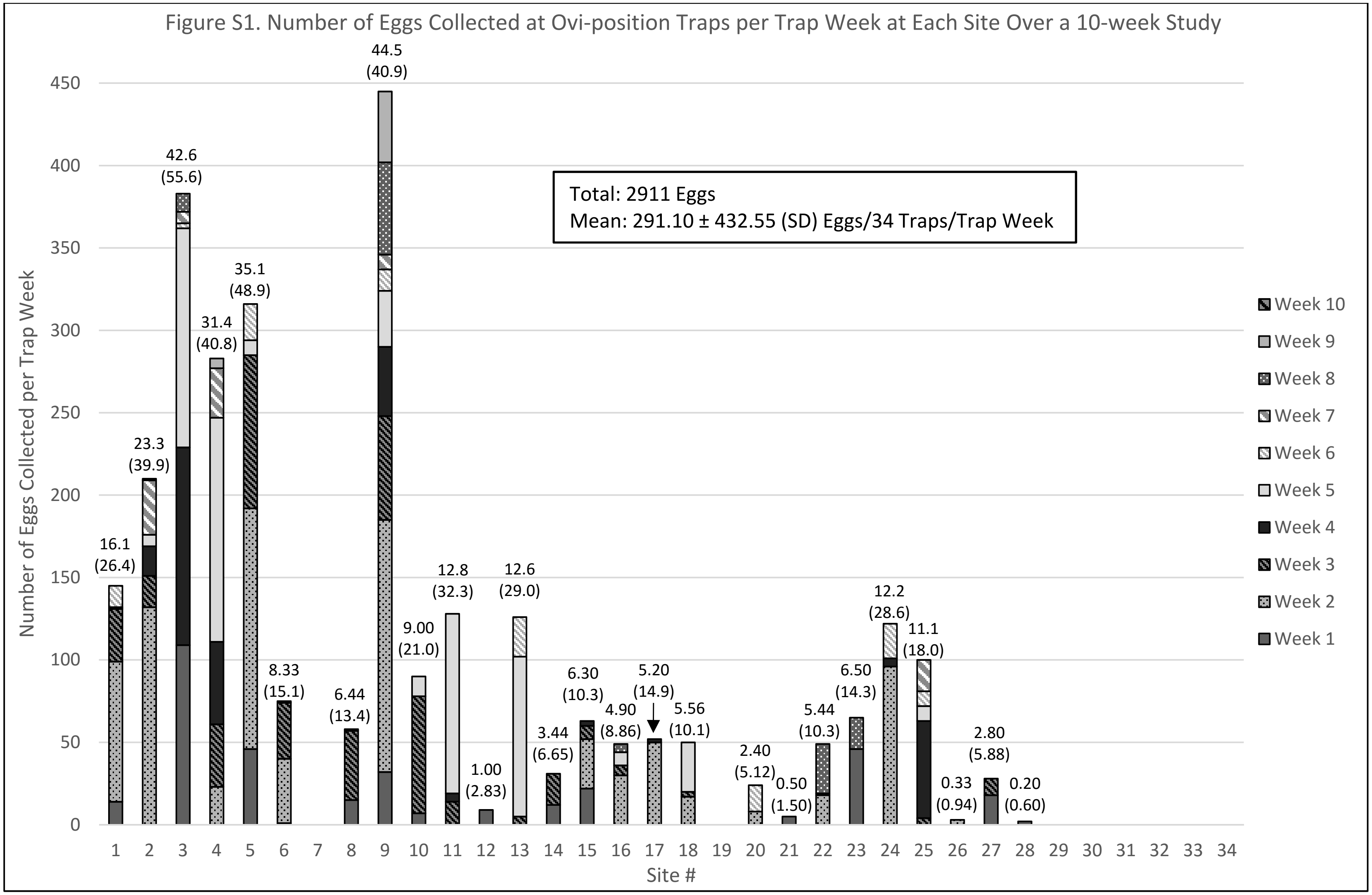

Fig. S1. Numbers above each bar represent the mean and (SD) for collection counts at that trap site across the ten weeks.

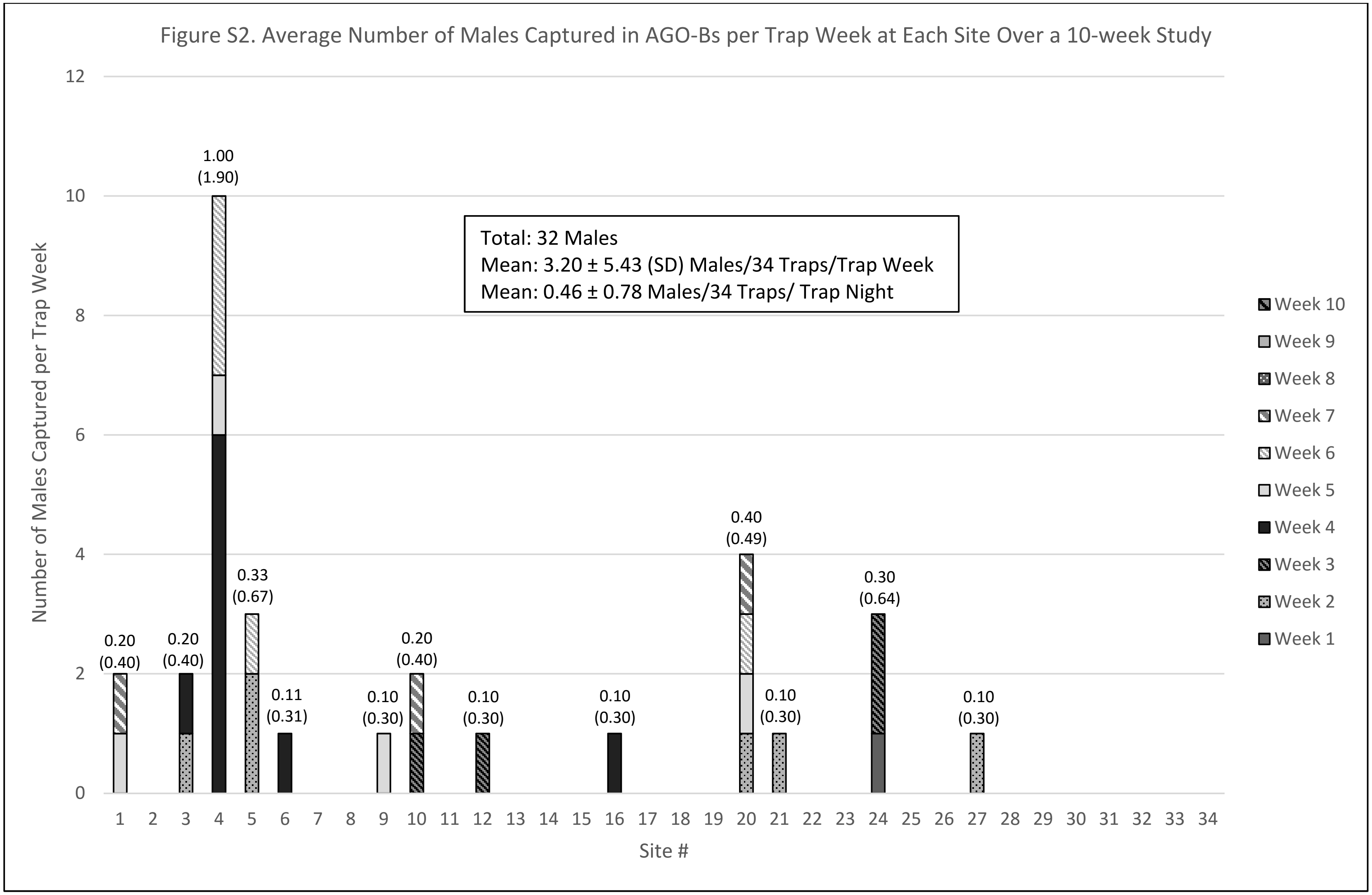

Fig. S2. Numbers above each bar represent the mean and (SD) for collection counts at that trap site across the ten weeks.

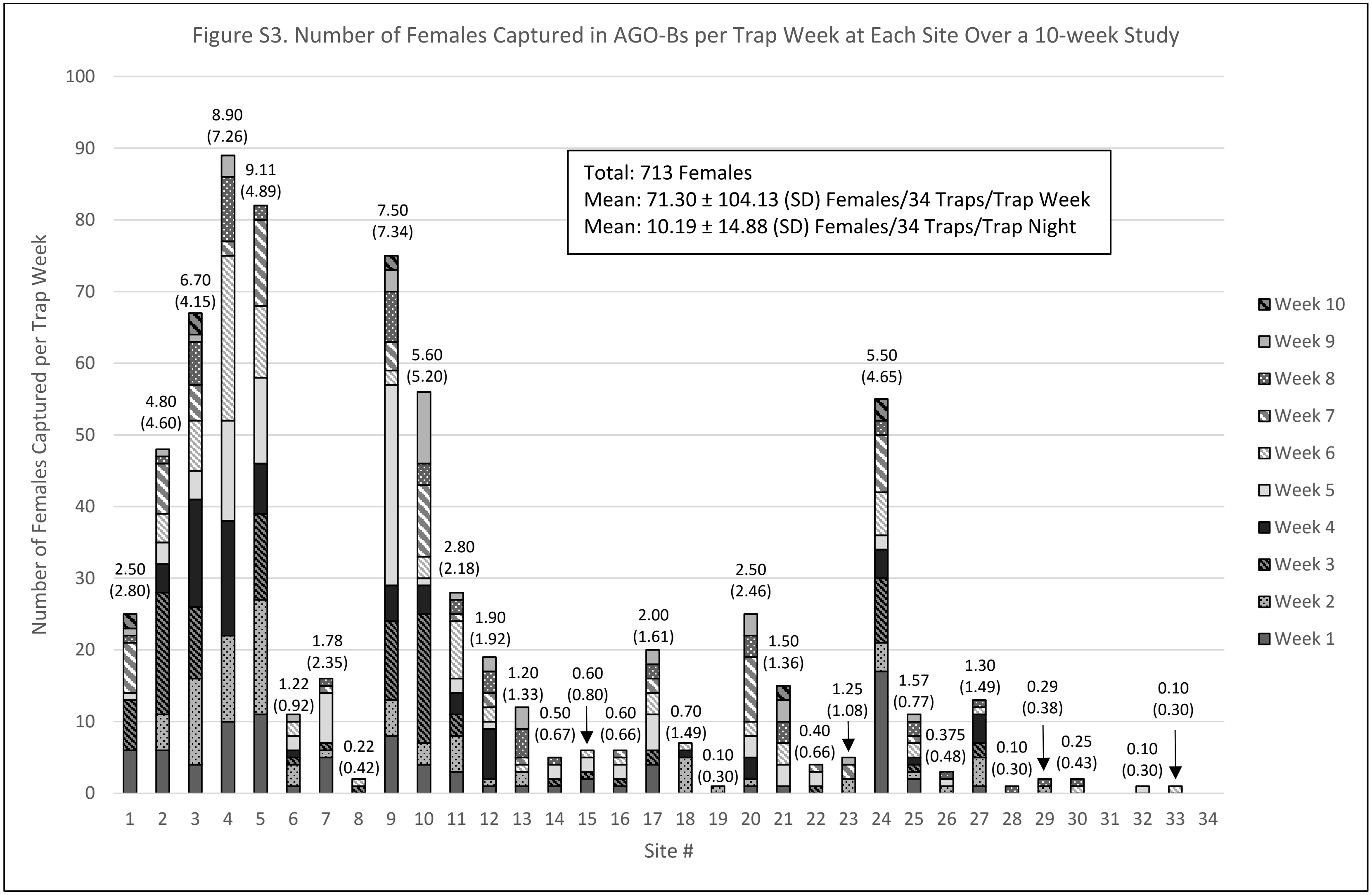

Fig. S3. Numbers above each bar represent the mean and (SD) for collection counts at that trap site across the ten weeks.

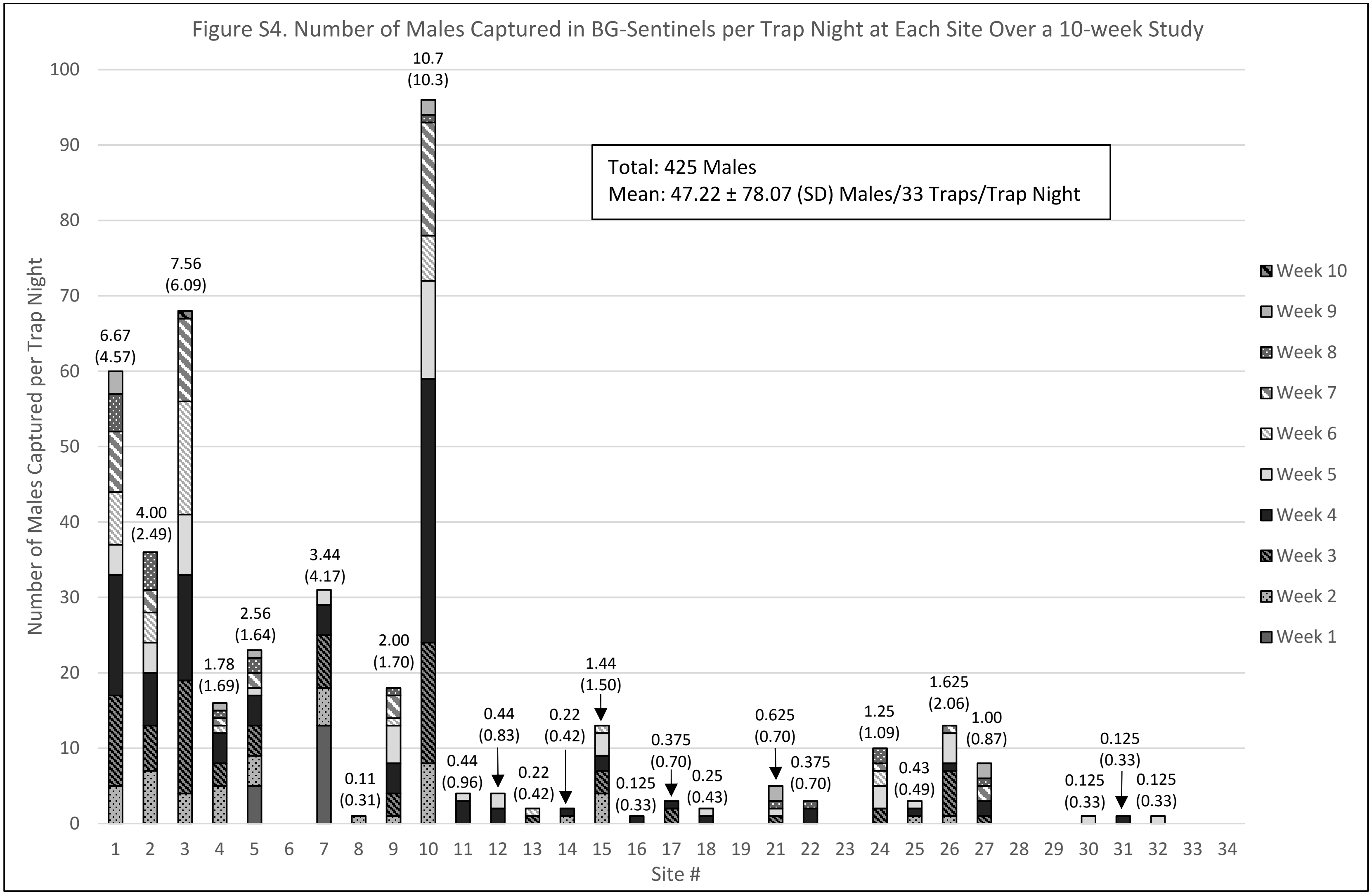

Fig. S4. Numbers above each bar represent the mean and (SD) for collection counts at that trap site across the ten weeks.

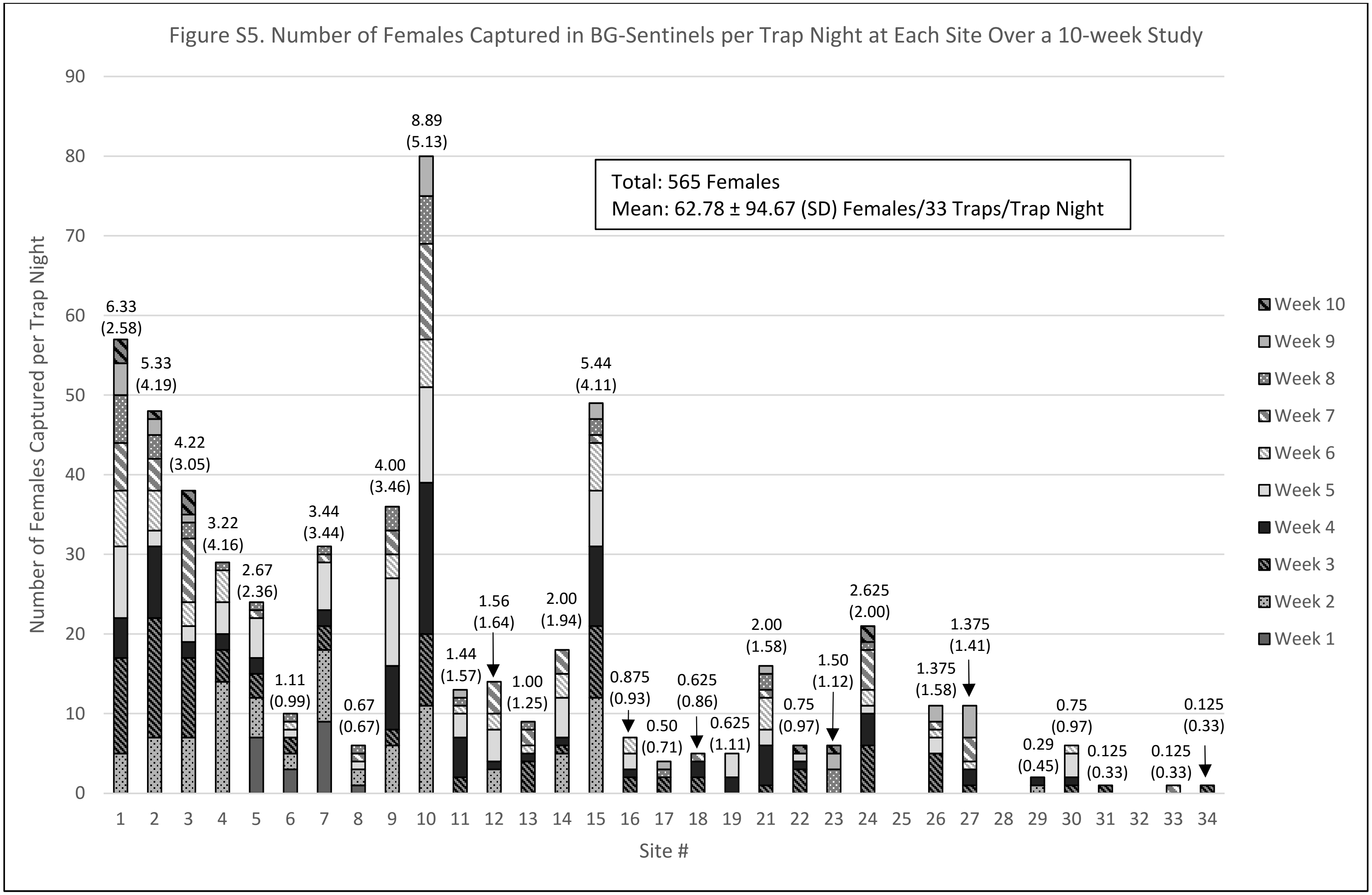

Fig. S5. Numbers above each bar represent the mean and (SD) for collection counts at that trap site across the ten weeks.

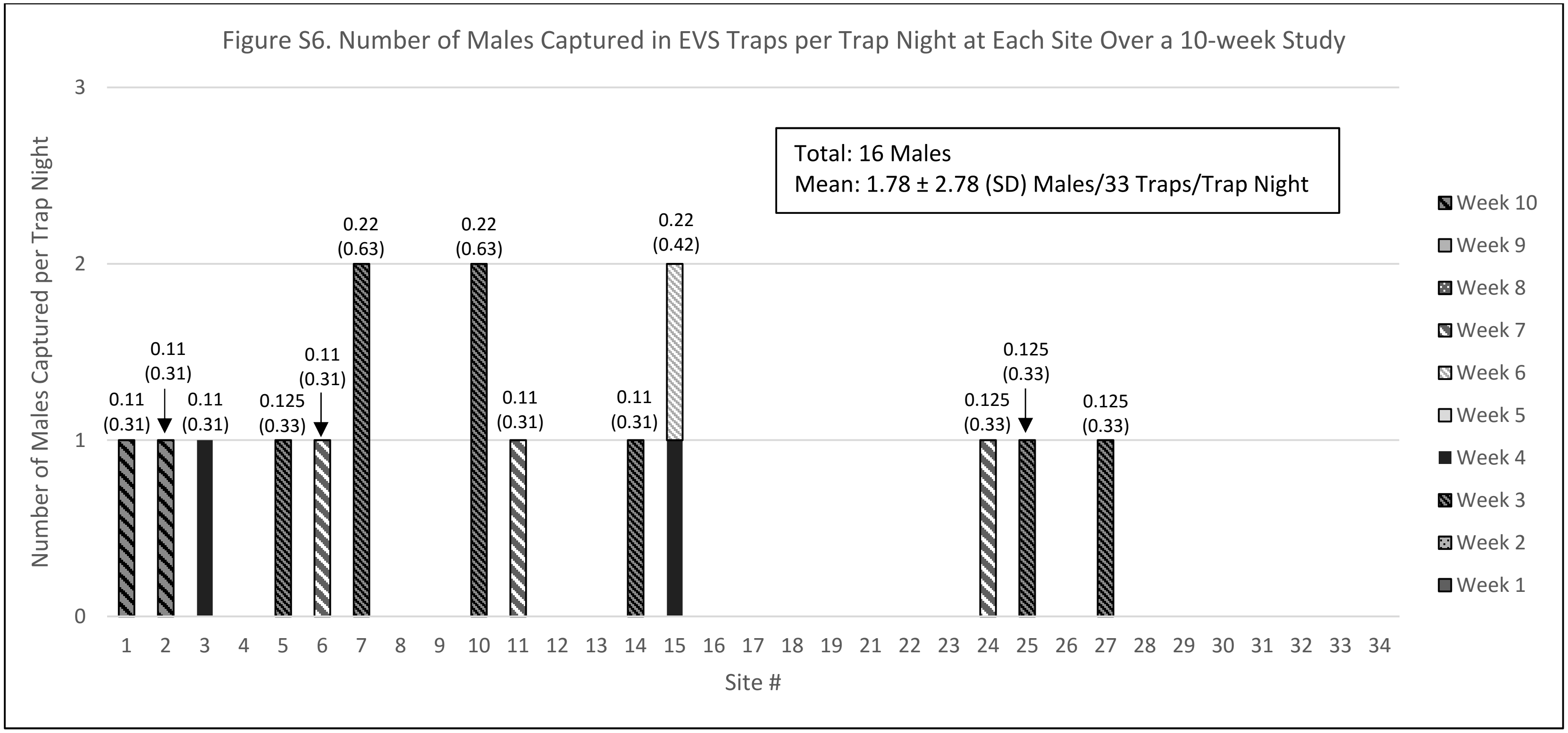

Fig. S6. Numbers above each bar represent the mean and (SD) for collection counts at that trap site across the ten weeks.

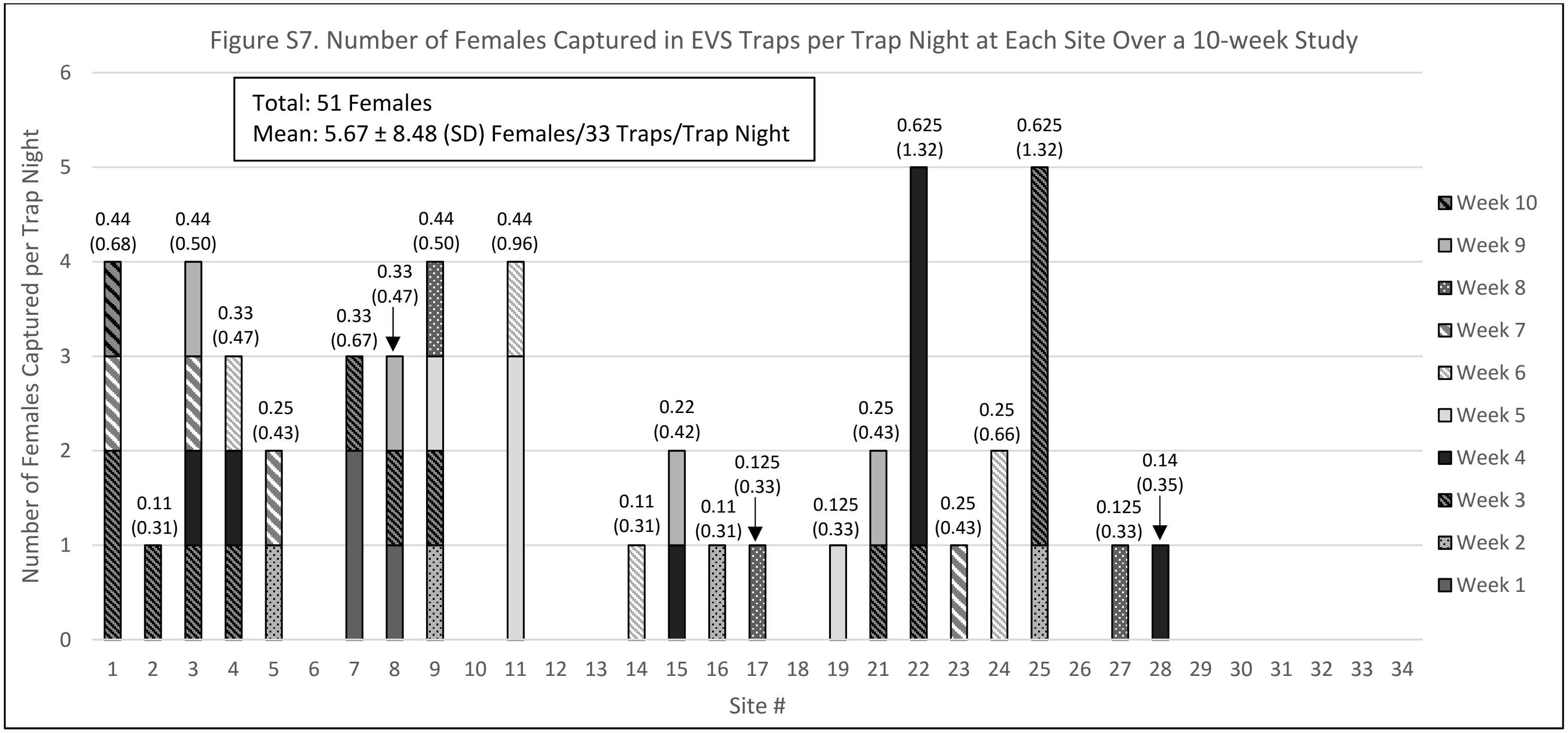

Fig. S7. Numbers above each bar represent the mean and (SD) for collection counts at that trap site across the ten weeks.

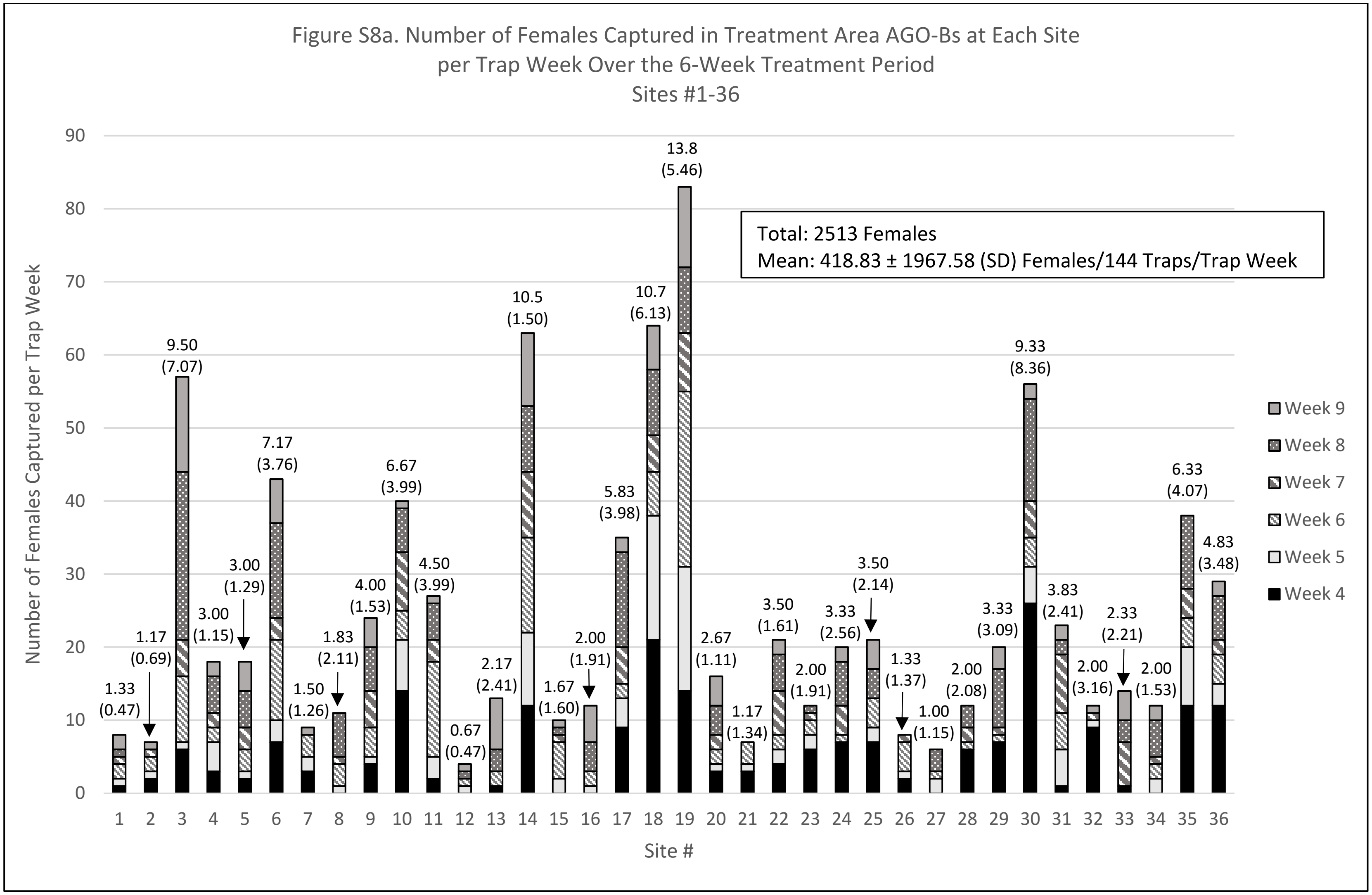

Fig. S8a. Numbers above each bar represent the mean and (SD) for collection counts at that trap site across the six weeks.

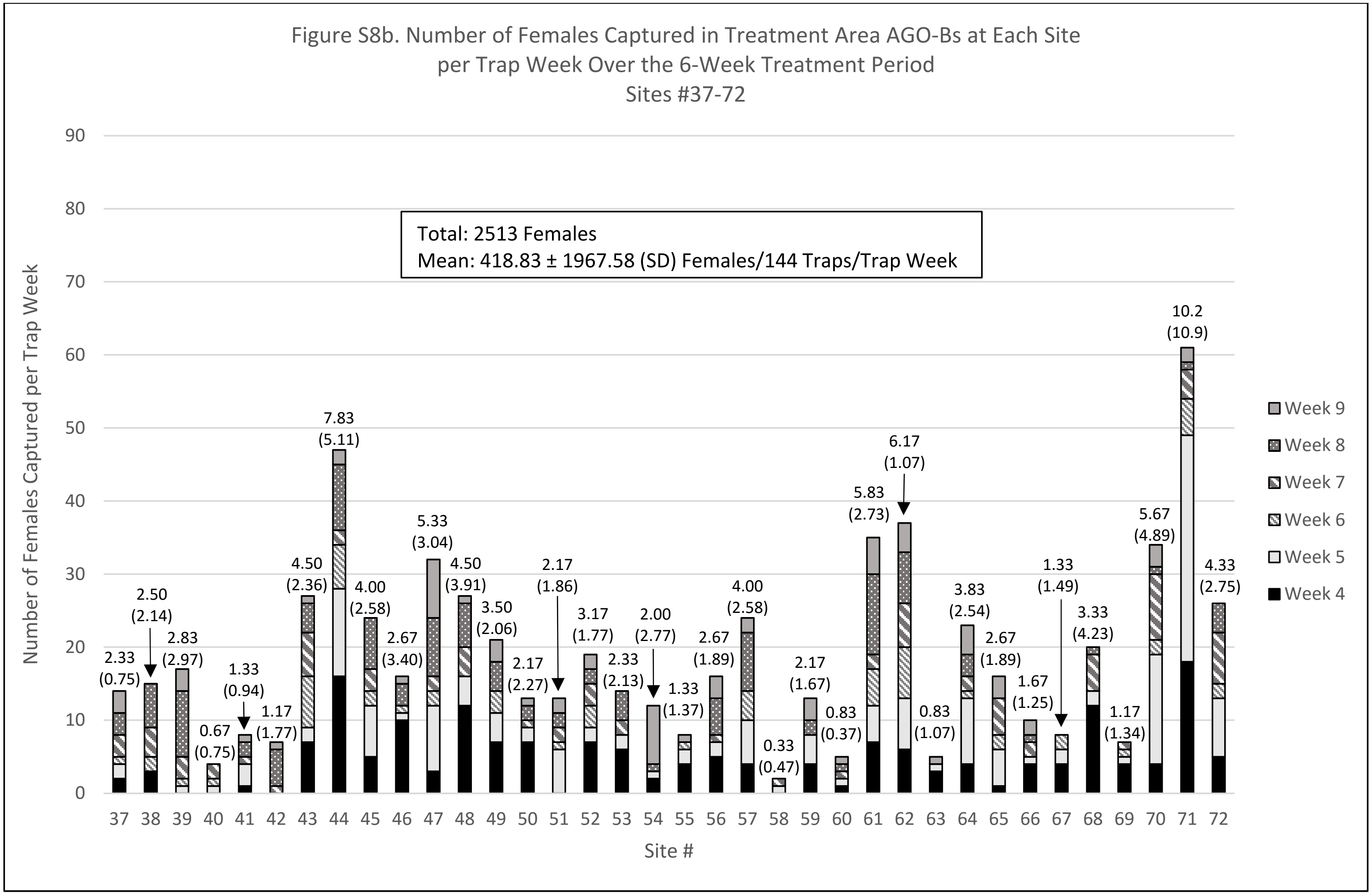

Fig. S8b. Numbers above each bar represent the mean and (SD) for collection counts at that trap site across the six weeks.

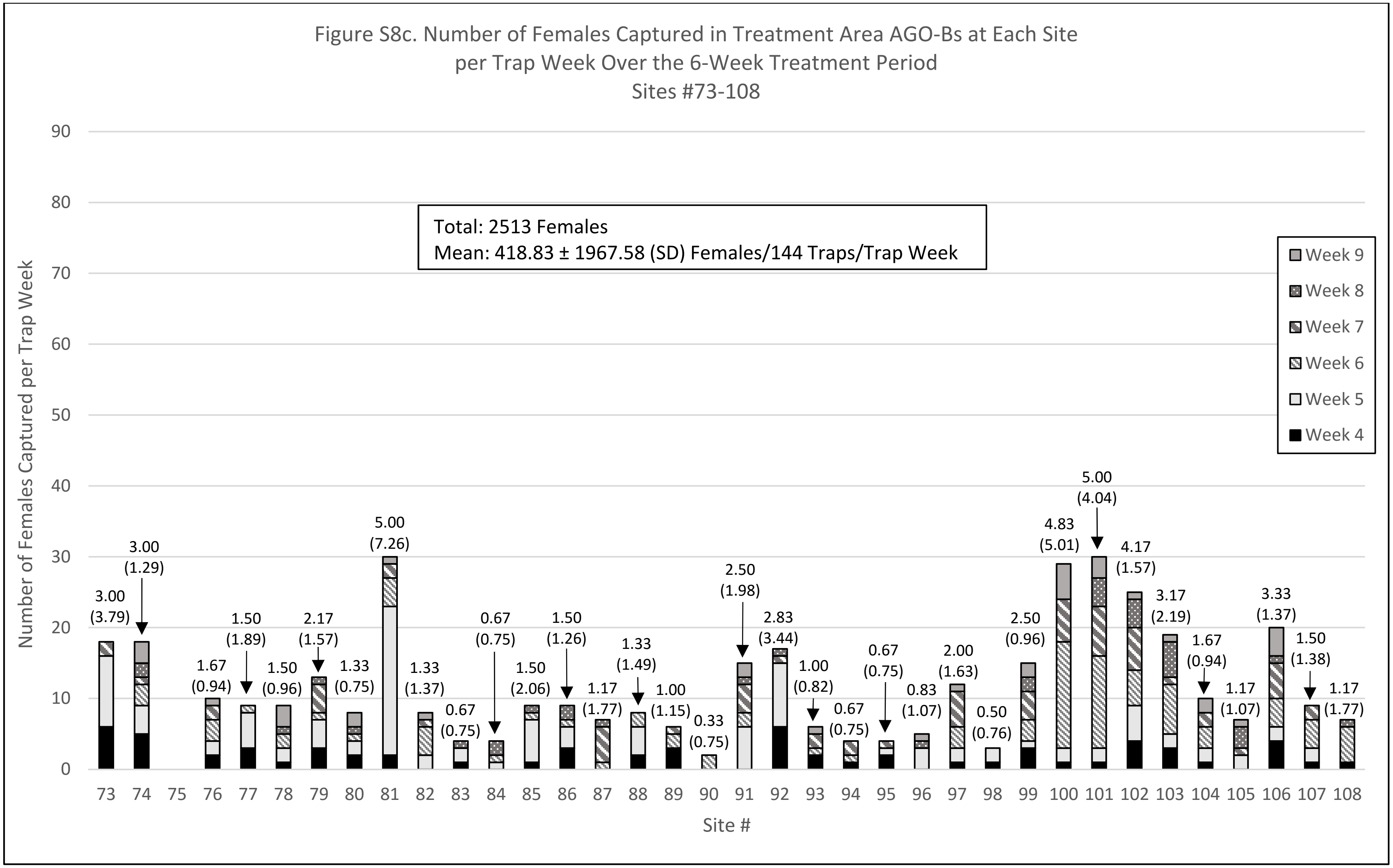

Fig. S8c. Numbers above each bar represent the mean and (SD) for collection counts at that trap site across the six weeks.

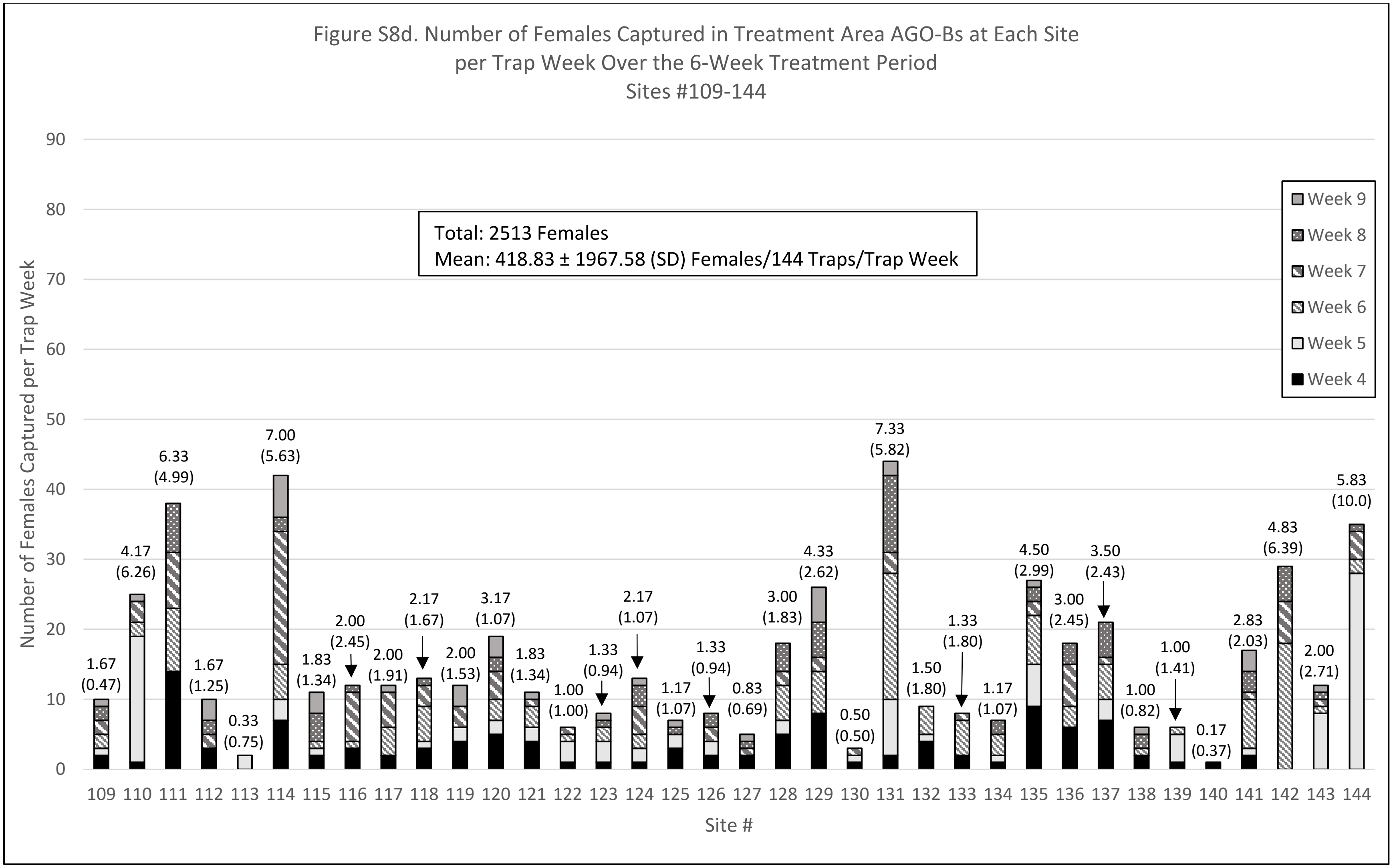

Fig. S8d. Numbers above each bar represent the mean and (SD) for collection counts at that trap site across the six weeks.

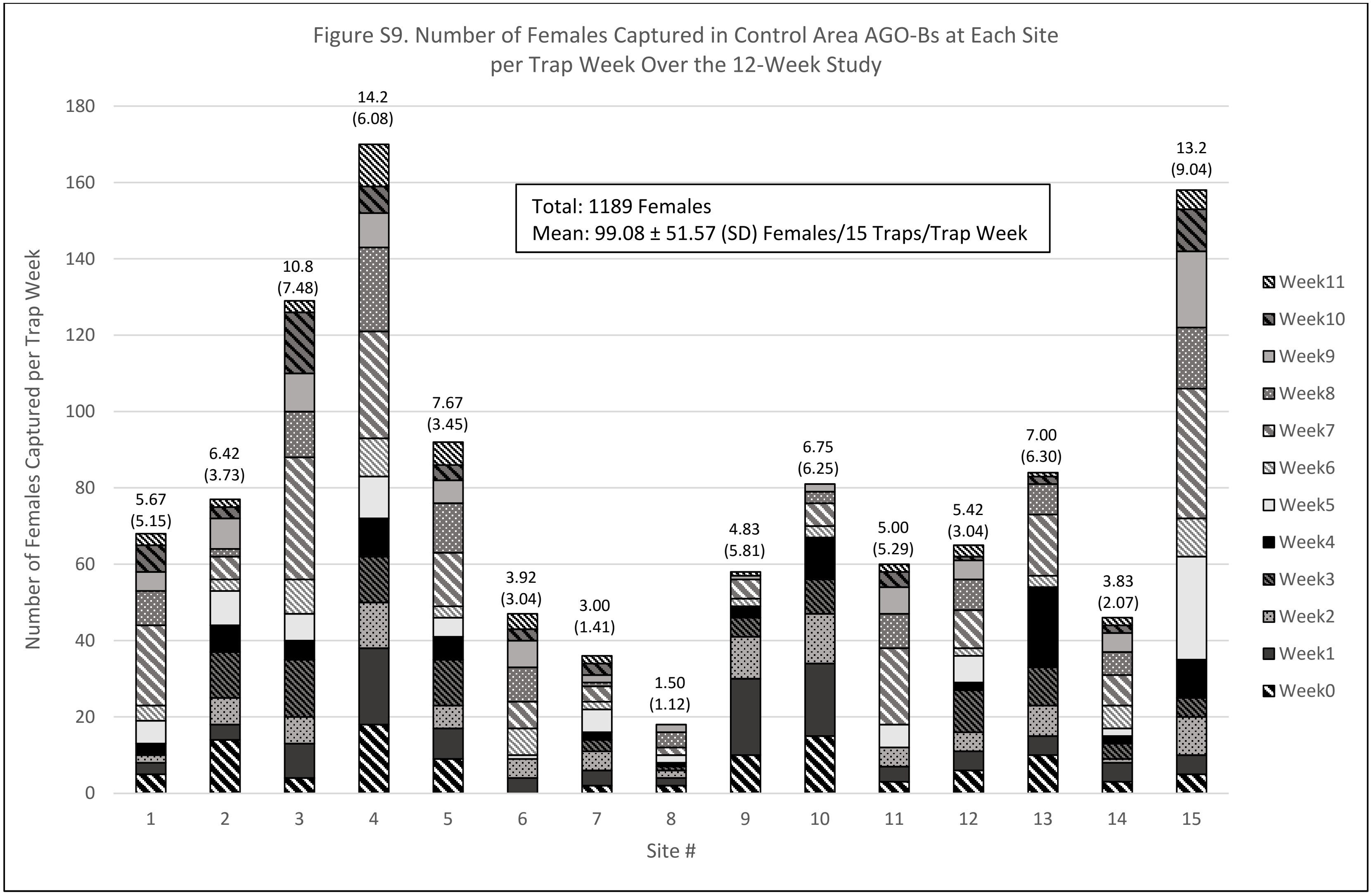

Fig. S9. Numbers above each bar represent the mean and (SD) for collection counts at that trap site across the twelve weeks.

Figure S10. BGS trap counts for AGO-B trap intervention trial. Red arrows indicate the date when AGO-Bs were deployed in the intervention area A. Blue shading in bars refer to male counts and orange refers to female counts. Yellow ring = 50m radius, Red = 100m radius and green ring = 200m radius.

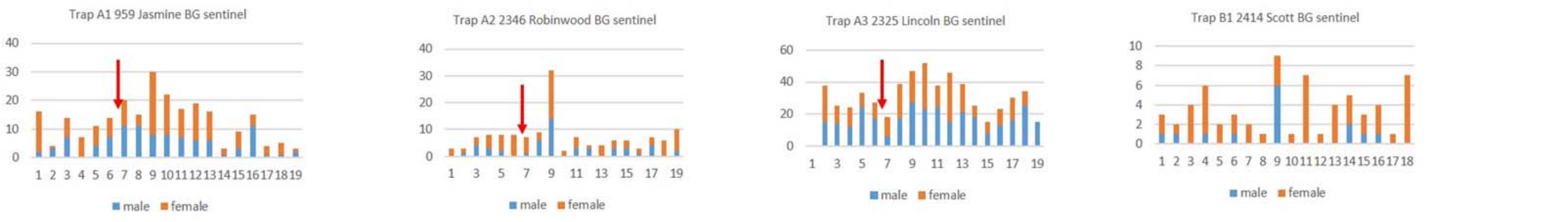

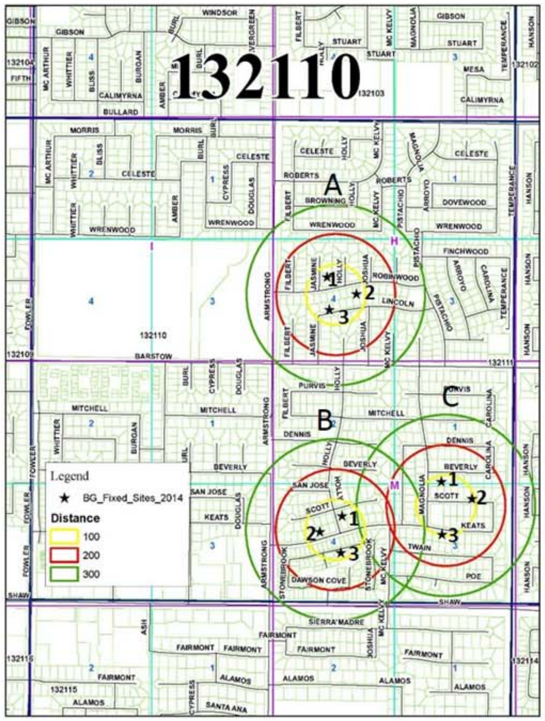

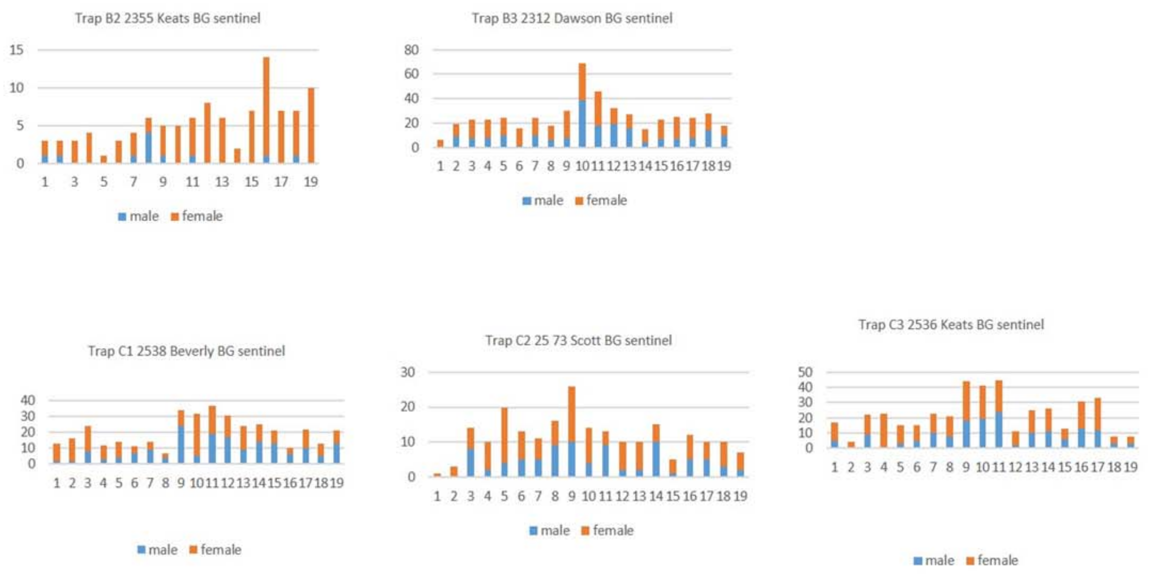

Trap night 1 (2-3 July). Trap night 2 () Trap night 5 (23 July 2014), Trap night 6 (25th July 2014), AGO set up on the 28th July 2014, Trap night 7 (30th July 2014) , day 8, trap night 9 (7th August 2014), trap night 10 (12th Aug 2014), trap night 11 (15th Aug 2014), trap night 12 (20th Aug 2014), trap night 13 (22 Aug 2014), trap night 14 (27th Aug 2014), trap night 15 (29th Aug 2014), trap night 16 (3rd Sept 2014), trap night 17 (6th Sept 2014), trap night 18 (10th Sept 2014), trap night 19 (12th Sept 2014).

Figure S11. The comparison of the IIS5-S6 region of the voltage gated sodium channel (*vgsc*) sequence between California *Aedes aegypti* and the reference *Aedes aegypti* sequence (Liverpool strain). Dark gray bar indicates exons, light gray bar for conservative domains, yellow box for single nucleotide polymorphisms observed or reported elsewhere, and the long yellow bar for the highly polymorphic intron regions. A light gray number above the reference sequence indicates the nucleotide position of the amplified product. Numbers next to small yellow box (a SNP) denote amino acid positions of the gene product. For example, S989P denotes the mutation at the 989th amino acid position from Serine (S) to Proline (P). The non-synonymous mutation that confer insecticide resistance (V1016I) is marked in red box.

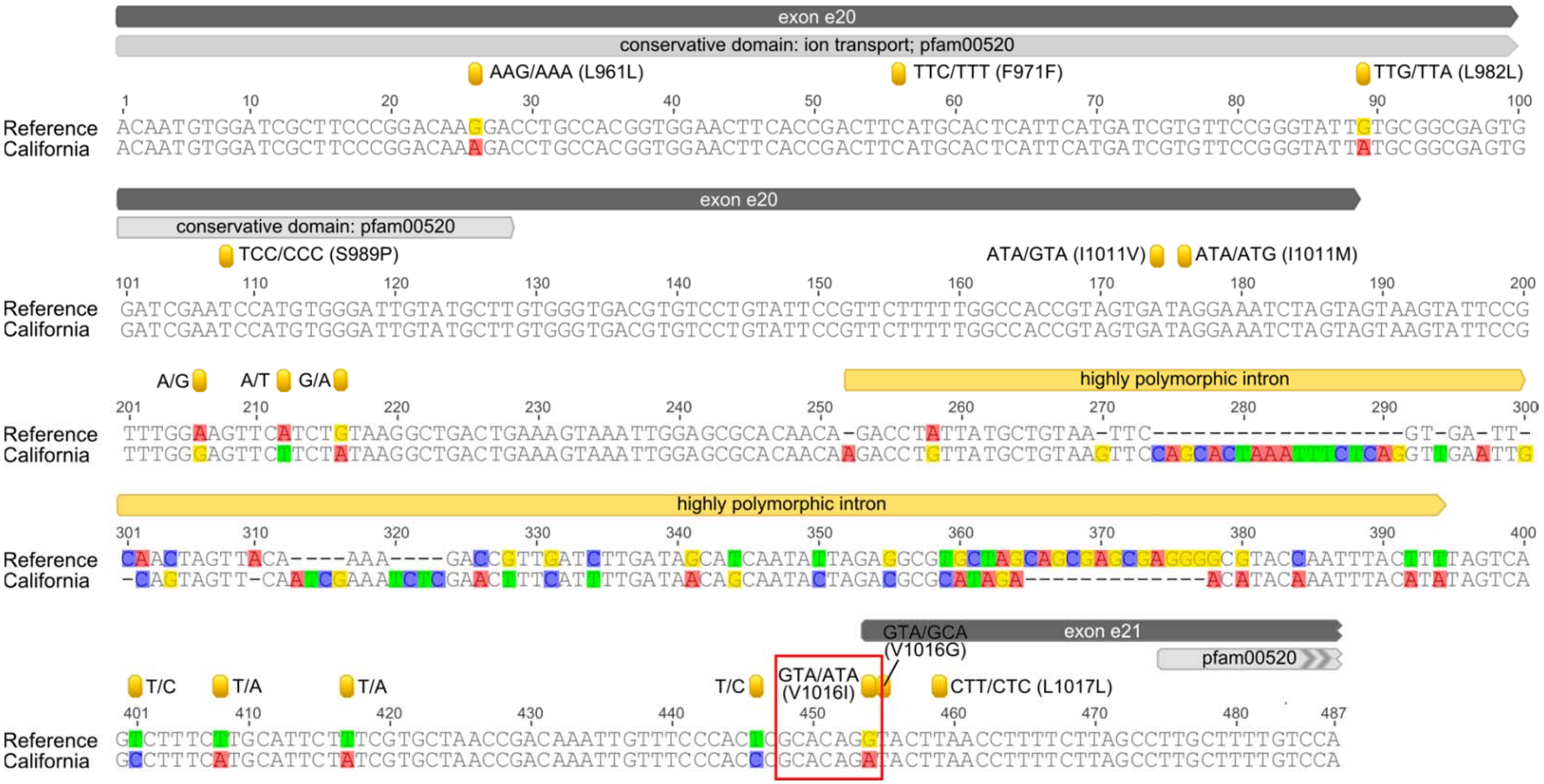

Supplement: Figures S1–S11 for ‘Surveillance, insecticide resistance and control of an invasive Aedes aegypti (Diptera: Culicidae) population in California’ [file f1000research-5-9305-s0000.tgz › de69dd5a-e74c-44bf-a35b-d35ac52557b3_Supplementary_Data_Figures_FINAL.pdf]
