# Supplementary material for: Surveillance, insecticide resistance and control of an invasive Aedes aegypti (Diptera: Culicidae) population in California
Source: F1000Res. 2016 Aug 5;5:194. Originally published 2016 Feb 19. [Version 3] doi: 10.12688/f1000research.8107.3 (PMC4857756; doi:10.12688/f1000research.8107.3)
Supplement: Peer review history

# Peer review: Referee response for version 2

**DOI:** 10.5256/f1000research.8879.r13399

**Published:** 2016-04-25

**Authors:** Saul Lozano-Fuentes

**Affiliations:** 1 Genesis Laboratories Inc, Wellington, CO, USA

My "reservations" have been addressed suitably. Thank you.

I have read this submission. I believe that I have an appropriate level of expertise to confirm that it is of an acceptable scientific standard.

# Peer review: Referee response for version 2

**DOI:** 10.5256/f1000research.8879.r13222

**Published:** 2016-04-06

**Authors:** Rui-De Xue

**Affiliations:** 1 Anastasia Mosquito Control District of St. Johns County, St. Augustine, FL, USA

It is a good article and provides a lot of useful information. The following suggestion and comments may assist the authors for further revision:

1. Add location GPS
2. Add blood resource and % sugar feeding for adult mosquitoes
3. Delete Culex from this paper even if it is a sensitive strain for comparison.
4. Add ULV truck speed and flowing rate.
5. Add the information about vegetation and any barrier objects for the trials in residential area.

I have read this submission. I believe that I have an appropriate level of expertise to confirm that it is of an acceptable scientific standard.

# Peer review: Referee response for version 1

**DOI:** 10.5256/f1000research.8722.r12576

**Published:** 2016-03-01

**Authors:** Saul Lozano-Fuentes

**Affiliations:** 1 Genesis Laboratories Inc, Wellington, CO, USA

I read this research with great enjoyment. The authors take a well-rounded approach to the surveillance and control of *Aedes aegypti*. However, I have a few methodological observations.

Methodologically, the calculation of the Knockdown Time (KDT) is commonly done using a binary logistic regression. From the text is unclear but I assumed that the authors are using the 50 ^th^ and 95 ^th^ percentiles for the estimation of the KDT _50_ and KDT _95_. Box plots are non-parametric; they are used without making any assumptions of underlying statistical distributions. The authors should make use of existing commonly used models to describe their time response observations. Several journals including, the Journal of Medical Entomology, request the use of logistic regression with a logit or probit link function. Using a common methodology allows the comparison of time response bioassay from different places and years. Thusly, please also provide estimates and confidence intervals for the KDT _50_ and KDT _90_ values using a binary logistic regression. Values beyond the KDT _90_ are outside the linear portion of the regression. This publication could help you quickly calculate these statistics Lozano-Fuentes *et al* ^1^.; other options exist in prepackage solutions like the “DRC” r library ( https://cran.r-project.org/web/packages/drc/drc.pdf)

Regarding the V1016I mutation, the authors provide evidence that the mutation is fixed in their samples and it is very likely in at a high frequency in the local population. Please add Vera-Maloof *et al*. ^2^ to your discussion to help frame this discovery with closer *Ae. aegypti *populations.

Regarding the AGO-Bs trap evaluation, the provided evidence (Figure 6, panel D) does not show a significant difference in mean relative abundance between any of the weeks as shown by the overlapping confidence intervals, some of the intervals are large enough that they appear to be below zero. Then again, the authors present a significant negative slope. These conflicting results point to the variables not being normal or not having equal variances. Please provide evidence that the data does not break the underlying assumptions of the linear regression model since it is being used to support the claim of abundance reduction by the AGO-B.

Other obsevations

Please add the citation to the R software to your manuscript ^3^.

I have read this submission. I believe that I have an appropriate level of expertise to confirm that it is of an acceptable scientific standard, however I have significant reservations, as outlined above.

## References

[1] Lozano-FuentesSSaavedra-RodriguezKBlackWCEisenL: QCal: a software application for the calculation of dose-response curves in insecticide resistance bioassays. *J Am Mosq Control Assoc*.2012;28(1) : 10.2987/11-6192.1 59-61 10.2987/11-6192.1 22533088

[2] Vera-MaloofFZSaavedra-RodriguezKElizondo-QuirogaAELozano-FuentesSBlack IvWC: Coevolution of the Ile1,016 and Cys1,534 Mutations in the Voltage Gated Sodium Channel Gene of Aedes aegypti in Mexico. *PLoS Negl Trop Dis*.2015;9(12) : 10.1371/journal.pntd.0004263 e0004263 10.1371/journal.pntd.0004263 26658798PMC4684211

[3] R Core TeamJ: R: A Language and Environment for Statistical Computing. *R Foundation for Statistical Computing*.2014; Reference source
